# Augmented reality adoption and the digital transformation of eco-smart tourism ventures: exploring the mediating role of tourist engagement

**DOI:** 10.1038/s41598-026-53572-7

**Published:** 2026-05-23

**Authors:** Muhammad Farhan Jalil, Azlan Ali, Wang Meiping

**Affiliations:** 1 Faculty of Economics and Business, Universiti Malaysia Sarawak, Jalan Dato Mohd Musa, 94300 Kota Samarahan, Malaysia; 2 City University, No. 8, Jalan 51A/223, Selangor Darul Ehsan, 46100 Petaling Jaya, Malaysia; 3 Guangdong University of Petrochemical Technology, No. 139, The Second Road Guandu, Maoming, Guangdong China

**Keywords:** Augmented reality, Tourist engagement, Digital transformation, Eco-Smart tourism, Small and medium enterprises, Business and management, Business and management, Information systems and information technology, Science, technology and society

## Abstract

The rapid growth of digital technologies has reshaped the tourism industry, with augmented reality (AR) emerging as a powerful tool for enhancing tourist experiences and driving business innovation. This study examines the role of AR adoption in fostering the digital transformation of eco-smart tourism ventures, with a particular focus on the mediating role of tourist engagement. Guided by the Stimulus–Organism–Response (S-O-R) theory, the research explores how AR adoption stimulates immersive experience, environmental awareness support, cultural enrichment, trust, perceived sustainability value, and innovation orientation, ultimately influencing tourist engagement and digital transformation. The study employed a quantitative research design, collecting data through structured questionnaires distributed to small and medium eco-smart tourism ventures across Malaysia. A total of 367 valid responses were analysed using AMOS - Structural Equation Modelling (AMOS-SEM) to test hypothesized relationships and mediation effects. The findings reveal that AR adoption significantly enhances immersive experience, environmental awareness, cultural enrichment, perceived sustainability value, and innovation orientation, while trust was found to be insignificant. AR adoption positively influences both tourist engagement and digital transformation, with tourist engagement serving as a complementary mediator between AR adoption and digital transformation. These results highlight the strategic role of AR not only as an immersive tool but also as a catalyst for sustainable digital transformation in tourism. The study contributes theoretically by extending the S-O-R framework into AR adoption research and practically by offering insights for tourism entrepreneurs and policymakers to leverage AR for innovation, sustainability, and competitiveness.

## Introduction

Tourism has long been recognized as one of the fundamental pillars of Malaysia’s economic and social development plan^1^. As a multicultural nation blessed with various natural sceneries and landscapes, a tapestry of cultural heritage, and rich biodiversity, Malaysia has situated tourism as a catalyst for global visibility and growth. The tourism sector contributes progressively to the national economy, causing around 14 to 15% of Malaysia’s GDP^2^. According to the Sun^3^, cited the findings of Department of Statistics Malaysia (DOSM), in 2023, the tourism sector contributed RM 275.8 billion, a sharp rebound from RM 251.5 billion in 2022, reflecting the sector’s resilience in overcoming the disturbances of the COVID-19 pandemic. According to Tourism Malaysia^4^, Malaysia received over 25 million international tourists in 2024, marking a 24.2% increase compared to 2023 and further strengthening its recovery momentum. According to The Nation-Thailand^5^, in early 2025, Malaysia had appeared as the top destination in Southeast Asia, recording 10.1 million foreign visitors in the first quarter of the year alone. These numbers highlight not only the financial weight of tourism but also its strategic role in achieving national development goals under the Malaysia Tourism Transformation Plan (MTTP) 2020 to 2030. Importantly, this strong growth trajectory also signals an increasing reliance on the sector’s capacity to adapt to digital transformation and sustainability imperatives, as global tourism markets become more experience driven, technology oriented, and environmentally conscious. In this regard, the competitiveness of Malaysia’s tourism sector is no longer determined solely by visitor numbers, but also by the extent to which tourism providers can integrate innovative technologies with sustainable practices to deliver value enhanced experiences.

Beyond economic indicators, the sector is also a major source of employment. In 2024, the World Travel and Tourism Council reported that the sector supported 357 million jobs worldwide, representing approximately 10% of the total global workforce. This includes both direct jobs such as tour operators, transport services, and accommodation providers and indirect jobs such as cultural performances, handicrafts, and local food supply. The scale of this contribution emphasizes tourism’s role as an inclusive industry that raises opportunities across urban and rural settings, thereby reducing income inequality and enhancing livelihoods. However, sustaining these socio-economic contributions increasingly depends on how effectively tourism stakeholders, particularly small and medium enterprises, respond to emerging digital and sustainability challenges that are reshaping the industry landscape. Despite the sector’s strong macro level performance, questions remain regarding the readiness of smaller tourism ventures to participate in this transformation, especially in terms of technological adoption and capability development. This highlights an important gap between national level tourism growth and firm level transformation capacity, which warrants further empirical investigation within the Malaysian context.

While large tourism companies often dominate headlines, small tourism enterprises, including boutique guesthouses, homestays, rural travel services, and community-based lodgings, form the backbone of visitor offerings across Malaysia’s diverse landscapes^6^. The importance of small tourism enterprises goes beyond their economic role. Small tourism embodies Malaysia’s commitment to dispersed tourism development, ensuring that tourism benefits are not confined to urban centres like Kuala Lumpur or Penang but extend into rural and underdeveloped regions^7^. They disseminate economic benefits locally while facilitating close knit, customized, and culturally genuine experiences. To encourage Indigenous led sustainable tourism, areas like Sarawak have embraced community-based tourism, such as the Sarawak Cultural Village. In decentralizing tourism benefits, small tourism enterprises promote cultural preservation^8^, reduce the risks of over tourism^9^, and encourage the adoption of eco-friendly practices^10^. According to Janjua et al.^11^, numerous small tourism businesses are already embracing sustainability concepts, such as reducing the use of single use plastics, encouraging eco-friendly lodging, and working with neighbourhood non-governmental organizations on environmental awareness campaigns. These characteristics position small tourism enterprises as key actors in advancing inclusive and sustainable tourism development, particularly in regions where community participation and environmental stewardship are critical to long term destination viability.

However, despite their vital role in sustaining Malaysia’s tourism landscape, small tourist businesses nevertheless face a number of operational and structural difficulties. These include inadequate digital capabilities^12^, limited access to financing^13^, barriers to scaling operations^14^, and insufficient international marketing exposure^15^. Such constraints often undermine their competitiveness against larger tourism players with stronger resources and global reach. At the same time, the tourism market is evolving rapidly, with travellers increasingly demanding eco-friendly, technology-enhanced, and personalized experiences^16^. This increasing expectation for sustainable and interactive offerings places further pressure on small tourism ventures to innovate.

In particular, eco-smart tourism ventures face a dual transformation challenge: they must simultaneously adopt advanced digital technologies while embedding sustainable practices into their core operations, often with limited resources and capabilities. In this context, Eco-Smart Tourism Ventures, small businesses that integrate sustainable (green) practices with smart technologies, emerge as a promising pathway forward^17^. These ventures not only safeguard the environment and local culture but also enhance the tourist journey through immersive and personalized engagement. This creates a critical tension between the increasing market demand for technology enabled and sustainable tourism experiences and the actual readiness of small tourism enterprises to deliver such offerings. Furthermore, the challenge is not only technological but also strategic, as small tourism operators often lack clear frameworks or guidance on how to integrate digital tools with sustainability objectives in a coherent and value creating manner^18,19^. This gap highlights the need for deeper empirical understanding of how emerging technologies can support the transformation of eco smart tourism ventures within resource constrained environments. Central to this transformation is the adoption of Augmented Reality (AR), which offers a powerful tool to bridge the innovation gap by enriching visitor experiences, promoting environmental awareness, and strengthening the competitive resilience of Malaysia’s small tourism enterprises.

AR has emerged as a powerful tool for reshaping the tourism sector by overlaying interactive digital content onto real-world environments^20^. Through AR, visitors can access improved storytelling, guided navigation, and deeper interpretation of cultural and ecological sites. In Malaysia, the adoption of AR and related immersive technologies such as Virtual Reality (VR) is steadily expanding^21^. For instance, applications like the SmartG travel app and interactive AR installations in museums and heritage sites have now started transforming how visitors involve with attractions. These technologies not only enhance visitor learning but also create more dynamic and memorable travel experiences. Nevertheless, existing research on AR in tourism has largely concentrated on urban destinations, large-scale tourism operators, or general user experience outcomes, with comparatively limited attention given to small and medium-sized eco-smart tourism ventures, particularly within developing country contexts such as Malaysia.

In ecologically sensitive and remote areas such as Sarawak, AR offers particular promise by supporting sustainable tourism practices. AR may offer virtual navigation aids, interpretive guides^22^, and eco-education platforms^23^, in place of building more infrastructure that can harm delicate ecosystems. This improves information accessibility while lessening the environmental effect. Furthermore, AR allows local communities to digitally preserve and showcase their cultural heritage^24^, making traditions, languages, and Indigenous knowledge more visible to a global audience^25^. Through embedding features such as multilingual narration and real-time eco-awareness prompts, AR strengthens both the economic opportunities and cultural identity of local communities. Moreover, AR has the ability to greatly increase visitor engagement by blending digital interaction with ecological responsibility, making trips not only more educational but also more impactful and unforgettable. However, the extent to which these technological affordances translate into measurable digital transformation outcomes for small eco-smart tourism ventures remains insufficiently explored in the current literature.

Tourist engagement serves as a crucial link between technological innovation and sustainable transformation in tourism. AR, with its immersive and interactive features, has the potential to significantly enhance this engagement^26^. When tourists are actively engaged, they are more likely to appreciate and internalize the value of eco-smart tourism practices^27^. This engagement not only heightens their satisfaction but also increases the likelihood of recommending the experience to others and returning for future visits. Such behaviours directly strengthen the business performance and green brand image of Eco-Smart Tourism Ventures. From a theoretical perspective, tourist engagement represents the ‘organism’ component within the Stimulus–Organism–Response (S-O-R) framework, capturing the internal cognitive and emotional states through which technological stimuli such as AR adoption may be associated with organisational outcomes. Therefore, tourist engagement functions as a vital mediating mechanism through which AR influences eco-smart transformation. Positioning engagement as a mediator allows for a more nuanced understanding of how AR adoption is linked with digital transformation, rather than assuming a direct effect, which remains underexplored in prior empirical studies. By motivating tourists to learn, participate, and connect meaningfully with destinations, engagement ensures that AR applications translate into long-term sustainability outcomes, benefiting both enterprises and the environment.

Despite the promise of AR in transforming tourism, many small tourism ventures still lack the awareness, resources, and confidence needed to adopt AR. Furthermore, empirical research exploring the role of AR in driving green transformation within Malaysian SMEs remains limited. While AR has been studied in broader contexts, its specific contribution to eco-smart tourism ventures, particularly in enhancing sustainability and resilience, has yet to be fully examined. Another critical gap lies in understanding the underlying mechanism of tourist engagement. Although AR is widely recognized for its immersive and interactive capabilities, little is known about how this engagement translates into meaningful outcomes such as environmental awareness, cultural preservation, and sustainable business performance. In addition, comprehensive frameworks and tailored strategies to guide small eco-smart tourism ventures in adopting AR effectively are scarce. Without such frameworks, these ventures risk falling behind in meeting the rising expectations for sustainability, digital interactivity, and personalized experiences. Accordingly, three key gaps emerge: (i) limited understanding of how eco-smart tourism ventures navigate digital and sustainability transformation challenges, (ii) insufficient empirical evidence on AR adoption within small tourism enterprises in Malaysia, and (iii) lack of clarity on the mediating role of tourist engagement in linking AR adoption with digital transformation outcomes. This study, therefore, aims to explore how AR adoption influences the digital transformation of eco-smart tourism small ventures, focusing on the mediating role of tourist engagement within the Malaysian small tourism context. Given the cross-sectional nature of the data, the study interprets these relationships as associative rather than strictly causal. Accordingly, we propose the following research questions:

### RQ1

How does AR adoption contribute to the digital transformation of eco-smart tourism ventures?

### RQ2

What role does tourist engagement play in mediating the relationship between AR adoption and digital transformation?

Grounded in the Stimulus–Organism–Response (S-O-R) theory, this study advances theoretical understanding by positioning AR adoption (stimulus) through six AR-specific dimensions, tourist engagement (organism) as the psychological and behavioural response, and the green digital transformation of eco-smart tourism ventures (response) in terms of image and performance. By doing so, it extends existing AR and tourism technology models with a sustainability-driven focus tailored to small ventures. This extension contributes to the growing body of literature integrating digital innovation with sustainability-oriented tourism research, particularly within SME and developing country contexts. Practically, the study provides actionable guidelines for small tourism businesses to implement AR in ways that strengthen their green branding and market competitiveness. It also supports policymakers and tourism boards, such as Tourism Malaysia, in designing training programs, subsidies, and digital infrastructure that enable AR-driven eco-smart ventures. Furthermore, the findings empower local communities and entrepreneurs to sustainably integrate cultural storytelling, environmental education, and digital innovation into their tourism offerings. These implications are particularly relevant for resource-constrained small tourism ventures seeking scalable and experience-driven pathways toward digital transformation.

## Literature review

### Stimulus–Organism–Response (S-O-R) theory

The Stimulus–Organism–Response (S-O-R) theory, originally developed by Mehrabian and Russell^28^, provides a robust lens through which to examine human behaviour in response to environmental stimuli. The model posits that external stimuli (S) influence individuals’ internal states (O), which in turn shape behavioural responses (R)^29^. In tourism and technology research, the S-O-R framework has been widely adopted to explain how digital innovations shape tourists’ cognitive, emotional, and behavioural patterns^30^. In situating AR adoption within this framework, the current study conceptualizes AR features as the “stimulus” that triggers tourist engagement (organism), ultimately leading to eco-smart transformation outcomes for small tourism ventures (response).

In this context, the stimulus refers to the adoption of AR technologies characterized by six specific dimensions such as immersive experience, environmental awareness support, cultural enrichment, trust, perceived sustainability value, and innovation orientation. These technological features provide the sensory and cognitive triggers that enhance tourists’ experiences, making them more memorable and engaging. The organism represents the tourists’ internal states, encompassing both psychological responses (e.g., emotional arousal, cognitive involvement, and environmental awareness) and behavioural engagement (e.g., participation, sharing, and advocacy). Finally, the response denotes the outcomes that emerge at the venture level, particularly the green transformation of eco-smart tourism enterprises, which may manifest in enhanced sustainability image, improved market competitiveness, and long-term performance.

Furthermore, the application of the S-O-R theory in this study extends its theoretical reach beyond traditional consumer behaviour studies into the domain of eco-smart tourism and sustainability. Previous research has largely applied S-O-R to retailing^31^, e-commerce^32^, and service environments to explain purchase intention, satisfaction, or loyalty^33^. By contrast, this study situates the framework within a sustainability-driven tourism context, offering a novel contribution to both tourism technology and green entrepreneurship literature. In particular, it demonstrates how digital technologies like AR can be strategically leveraged not only for enhanced customer experiences but also for advancing environmental education, cultural preservation, and sustainable business practices.

### AR and immersive experience

Immersive experience has emerged as one of the defining value propositions of AR in the tourism sector. The concept of immersion refers to the psychological state in which an individual feels deeply engaged, absorbed, and transported into an experience beyond ordinary reality^34^. Unlike conventional forms of digital communication, AR blends physical and virtual environments, allowing tourists to engage interactively with cultural, natural, or historical contexts. This fusion fosters a sense of presence and authenticity, enabling tourists to feel as though they are co participants rather than passive observers. In eco smart tourism ventures, immersive AR applications can overlay environmental narratives, historical reconstructions, or ecological insights directly onto physical settings, transforming ordinary visits into enriched, participatory learning experiences^35^.

The immersive nature of AR is built on three interrelated elements: interactivity, vividness, and personalization^36^. Interactivity allows users to operate or explore digital objects within real-world settings, stimulating active involvement and curiosity. Vividness refers to the richness of sensory input providing by AR applications, whether through 3D graphics, animations, or soundscapes, that amplify engagement and create unforgettable encounters. Personalization ensures that immersive content is aligned with tourists’ preferences, thereby enhancing relevance and emotional resonance. Collectively, these features not only intensify tourists’ experiential value but also support the perceived value of AR adoption for tourism providers who seek to deliver distinctive offerings in competitive markets.

From a theoretical perspective grounded in the S-O-R framework, AR adoption is conceptualized as a technological stimulus that shapes tourists’ internal experiential states. Within this framework, immersive experience represents an organism level response that reflects tourists’ cognitive and affective reactions to AR enabled interactions. This implies that the degree to which tourism ventures adopt and implement AR technologies is likely to influence the intensity of immersive experiences perceived by tourists^37^.

From a managerial perspective, rather than serving as a driver of AR adoption, immersive experience is better understood as a value outcome that tourism providers seek to achieve through the implementation of AR technologies. Tourism operators are increasingly aware that delivering immersive experiences is central to creating differentiation, enhancing tourist satisfaction, and encouraging repeat visitation^38^. Through integrating immersive AR applications, tourism ventures can enhance service innovation, elevate their sustainability profile, and appeal to tourists who increasingly demand experiential, tech-enabled, and eco-conscious travel options. Hence, immersive experience as a key mechanism through which AR adoption contributes to enhanced service delivery and experiential differentiation in competitive tourism markets.

Empirical studies highlight the role of immersive experiences in shaping technology adoption decisions. Dağ et al.^39^ reported that AR features such as interactivity and realism significantly enhance users’ sense of immersion and experiential engagement. Dalmazi et al.^40^ found that AR based environments create more engaging and satisfying experiences by deepening users’ psychological involvement. Similarly, Jiang et al.^41^ emphasized that AR applications in heritage and eco-tourism contexts enhance immersive experiences by enabling interactive and context rich storytelling. These findings collectively suggest that immersive experience is an outcome of AR adoption, reinforcing its positioning as an organism level response within the S-O-R framework. Accordingly, this study conceptualizes immersive experience as a direct outcome of AR adoption in eco smart tourism ventures, reflecting tourists’ internal experiential responses to AR enabled environments. Therefore, the following hypothesis is proposed:

#### Hypothesis H1

AR adoption has a significant positive relationship with Immersive experience.

### AR and environmental awareness support

Environmental awareness has become a central driver in shaping consumer behaviour and business strategies in the tourism sector. Increasingly, tourists demand sustainable practices that align with ecological responsibility, cultural preservation, and green innovation^42^. For small eco-smart tourism ventures, the ability to communicate environmental values effectively is essential to creating competitive advantage. However, traditional forms of awareness campaigns—such as brochures, posters, or guided talks—are often limited in their ability to engage tourists meaningfully. Here, AR emerges as a transformative tool that enhances environmental awareness support by providing interactive, real-time, and immersive learning experiences.

AR can facilitate environmental education by overlaying digital information, narratives, or simulations directly onto natural or cultural sites^43^. For example, tourists visiting eco-parks or heritage sites can use AR-enabled devices to visualize endangered species in their natural habitats, understand the ecological impact of human activity, or explore sustainable practices adopted by local communities. This digital augmentation transforms environmental knowledge from passive information into active, engaging experiences. By doing so, AR not only enhances tourists’ awareness of ecological and cultural sustainability but also fosters greater empathy and responsibility toward protecting the environment.

From a theoretical perspective grounded in the S-O-R framework, AR adoption is conceptualized as a technological stimulus that shapes tourists’ internal cognitive and affective states. Within this framework, environmental awareness support represents an organism level response, reflecting the extent to which tourists develop heightened understanding, concern^44^, and sensitivity toward environmental sustainability as a result of AR enabled experiences.

From the perspective of small tourism ventures, AR-driven environmental awareness provides a dual benefit. On one hand, it educates and influences tourists to adopt sustainable behaviours, thereby enhancing the overall eco-tourism experience^45^. On the other hand, it strengthens the green brand identity of the business, signalling its commitment to environmental stewardship^46^. This alignment with sustainability trends can attract eco-conscious travellers, generate positive word-of-mouth, and improve customer loyalty. Furthermore, AR-driven awareness initiatives resonate strongly with global movements such as the United Nations Sustainable Development Goals (SDGs), particularly Goal 12 (responsible consumption and production) and Goal 13 (climate action), making them highly relevant for small ventures aiming to remain competitive in the global tourism landscape. In this sense, environmental awareness support is not a precursor to AR adoption but rather a strategic outcome that tourism providers aim to achieve through the effective implementation of AR technologies.

Empirical studies have supported the link between AR applications and increased environmental awareness. For instance, research has shown that AR enhances tourists’ cognitive engagement with sustainability matters, leading to improved knowledge retention and environmentally responsible behaviours^47,48^. Similarly, Ercan et al.^49^ found that AR-supported eco-tourism experiences promoted stronger pro-environmental attitudes among visitors, compared to traditional interpretative methods. These findings collectively indicate that environmental awareness support emerges as an outcome of AR adoption, reinforcing its role as an organism level response within the S-O-R framework. Accordingly, this study conceptualizes environmental awareness support as a direct outcome of AR adoption in eco smart tourism ventures, reflecting tourists’ enhanced understanding and responsiveness toward sustainability issues. Therefore, we propose the following hypothesis:

#### Hypothesis H2

AR adoption has a significant positive relationship with environmental awareness support.

### AR and cultural enrichment

Cultural enrichment is a central element of tourism experiences, particularly in eco-smart and heritage-based ventures, where travellers seek not only leisure but also meaningful encounters with local traditions, histories, and identities. In the context of tourism, cultural enrichment refers to the extent to which visitors are exposed to and engaged with the cultural heritage, practices, and stories of a destination^50^. Traditionally, cultural enrichment has been facilitated through guided tours, museums, folklore performances, and community engagement. However, such methods often suffer from limitations of accessibility, scalability, and depth of interactivity. AR has emerged as a powerful tool to address these limitations by enhancing tourists’ cultural learning experiences in dynamic and interactive ways.

AR provides opportunities for cultural storytelling by overlaying digital narratives, 3D reconstructions, and interactive features onto physical heritage sites^51^. For example, AR applications can allow tourists to witness historical events re-enacted at archaeological ruins, visualize traditional crafts in 3D before their eyes, or explore intangible cultural heritage, such as folklore, myths, or rituals, in ways that blend the physical environment with immersive digital overlays. AR enables travellers to not only observe culture but to actively participate in it, creating a sense of deeper connection and engagement with the host community^52^. Such experiential enrichment resonates strongly with modern tourists, especially younger generations who value authenticity and technological integration in their travel experiences.

From a theoretical perspective grounded in the S-O-R framework, AR adoption is conceptualized as a technological stimulus that shapes tourists’ internal experiential states. Within this framework, cultural enrichment represents an organism level response, reflecting tourists’ cognitive and affective engagement with cultural content^53^, as facilitated through AR enabled interactions.

From the perspective of eco-smart tourism ventures, AR-driven cultural enrichment offers a way to differentiate their services and strengthen value propositions. By embedding cultural education within AR platforms^54^, small ventures can both preserve and promote local traditions, ensuring that cultural knowledge is transferred across generations while simultaneously enhancing the visitor experience. Moreover, cultural enrichment through AR contributes to sustainable tourism development by reducing physical strain on heritage sites^48^. Instead of large groups crowding fragile historical monuments, AR can provide digital reconstructions and simulations, thereby protecting cultural assets while still delivering rich educational value. In this regard, cultural enrichment is more appropriately understood as a value outcome achieved through AR implementation rather than a precursor to its adoption.

Empirical evidence supports the role of AR in cultural enrichment within tourism contexts. Studies have shown that AR applications significantly enhance tourists’ cultural learning and appreciation, particularly in heritage and museum tourism^52,55^. Han et al.^56^ reported that AR-supported cultural experiences improved both tourists’ satisfaction and their perception of cultural authenticity. Similarly, Wen et al.^57^ found that AR technologies enhanced tourists’ cultural engagement and experiential value by bridging modern digital interfaces with heritage-based content. These findings collectively indicate that cultural enrichment emerges as an outcome of AR adoption, reinforcing its role as an organism level response within the Stimulus Organism Response framework rather than as an antecedent. Accordingly, this study conceptualizes cultural enrichment as a direct outcome of AR adoption in eco smart tourism ventures, reflecting tourists enhanced cultural understanding and experiential engagement. Thus, we propose the following hypothesis:

#### Hypothesis H3

AR adoption has a significant positive relationship with cultural enrichment.

### AR and trust

Trust is a cornerstone of technology adoption in tourism, as travellers often rely on digital solutions to guide, inform, and enhance their experiences^58^. In the context of Augmented Reality (AR), trust refers to tourists’ confidence in the reliability, credibility, and usefulness of AR applications in delivering accurate information, protecting their privacy, and ensuring seamless functionality^59^. Since AR integrates digital overlays into real-world settings, tourists must perceive the system as dependable, safe, and free of misleading or distorted content. Without sufficient trust, even the most innovative AR applications risk rejection by potential users.

In tourism experiences, trust plays multiple roles. First, tourists must trust the accuracy and authenticity of the information delivered through AR applications^60^. For example, when AR platforms display historical reconstructions or eco-environmental information about a site, visitors need to feel assured that the content is factually correct and not exaggerated for commercial purposes. Second, trust extends to the security and ethical use of tourists’ personal data^61^. AR applications often utilize GPS tracking, location-based services, and sometimes even biometric inputs, raising concerns about data privacy. Therefore, perceived security and confidentiality form part of the trust equation. Finally, trust encompasses system performance^62^, travellers are more likely to adopt AR when they believe the system will function smoothly without technical glitches, interruptions, or inaccuracies that could compromise their overall experience.

From a theoretical perspective grounded in the S-O-R framework, AR adoption is conceptualized as a technological stimulus that shapes tourists’ internal perceptions and psychological states^63^. Within this framework, trust represents an organism level response, reflecting tourists’ confidence in the reliability, security, and credibility of AR enabled experiences as a result of their interaction with the technology.

Meanwhile, an eco-smart tourism perspective, trust is vital because these ventures often depend on limited resources to promote sustainability, authenticity, and innovation. In this context, trust is not a precursor to AR adoption but an experiential outcome that emerges when AR technologies are effectively implemented and consistently deliver accurate, secure, and reliable interactions. According to Wang et al.^64^, AR adoption is perceived as manipulative, unreliable, or untrustworthy, it could undermine tourists’ engagement and damage the reputation of eco-smart ventures. Conversely, establishing trust in AR applications enhances tourists’ willingness to use them, thereby strengthening both their engagement and satisfaction^65^. Trust also contributes to a sense of psychological comfort: when travellers feel confident in AR tools, they are more open to experimenting with novel ways of experiencing culture, environment, and eco-sustainability narratives.

Empirical research underlines the importance of trust in technology adoption. Vorm et al.^66^ demonstrated that trust is a central factor in determining user acceptance of new technologies, especially when there is uncertainty about performance or data privacy. In tourism, Chen et al.^67^ found that travellers’ trust in AR applications positively affected their intention to use AR for heritage and cultural tourism. Similarly, Xu et al.^68^ reported that trust in AR technologies was significantly related to tourists’ perceptions of usefulness and ease of use, which in turn influenced adoption. Moreover, Alimamy and Nadeem^69^ suggest that trust in AR contributes to higher satisfaction and stronger engagement, particularly in contexts where cultural and environmental authenticity are crucial. Accordingly, this study conceptualizes trust as a direct outcome of AR adoption in eco smart tourism ventures, reflecting tourists’ confidence in the reliability and integrity of AR enabled experiences. Therefore, we propose the following hypothesis:

#### Hypothesis H4

AR adoption has a significant positive relationship with trust.

### AR and perceived sustainability value

Perceived sustainability value refers to the extent to which tourists recognize and appreciate the environmental, social, and cultural benefits of adopting a particular innovation or practice^70^. In the tourism context, it captures the degree to which tourists believe that their travel experiences or behaviours contribute to eco-friendly practices, resource efficiency, and the preservation of cultural and natural heritage. With increasing global awareness of climate change and sustainability, tourists are not only seeking memorable experiences but also evaluating whether their choices align with sustainable values^71^. This alignment becomes critical for eco-smart tourism ventures, which emphasize environmental responsibility and cultural authenticity.

AR provides a unique platform to communicate and enhance perceived sustainability value in tourism. Unlike traditional information delivery, AR immerses tourists in interactive, real-time experiences that demonstrate eco-sustainability initiatives^72^. For instance, AR applications can illustrate the ecological footprint of local practices, simulate the environmental benefits of conservation, or recreate endangered habitats to raise awareness. Through such interactive storytelling, tourists can gain a deeper understanding of how their travel contributes to sustainability goals. When travellers perceive that AR, applications help them learn about and support sustainable tourism practices^48^, their perceived sustainability value increases, making them more inclined to adopt AR as part of their journey. In this sense, AR functions as a mechanism through which sustainability related information is not only communicated but also experienced, thereby strengthening tourists’ perception of sustainability value.

From a theoretical perspective grounded in the S-O-R framework, AR adoption is conceptualized as a technological stimulus that shapes tourists’ internal evaluative and perceptual states. Within this framework, perceived sustainability value represents an organism level response, reflecting tourists’ cognitive assessment of the environmental and social benefits derived from AR enabled tourism experiences^73^. From a managerial perspective, perceived sustainability value is a strategic outcome that eco smart tourism ventures aim to achieve through the implementation of AR technologies. By enhancing tourists’ understanding of sustainability practices, AR enables ventures to strengthen their green positioning and create value driven experiences that align with the expectations of environmentally conscious travellers^74^.

Empirical research supports the relationship between perceived sustainability value and technology adoption. For example, Sharma et al.^75^ found that consumers’ perception of green value directly influences their willingness to adopt eco-friendly innovations. In the tourism domain, Sujood et al.^76^ reported that travellers perceived environmental and social value significantly impacts their intention to engage with smart and green tourism technologies. Similarly, study by Yu et al.^77^ demonstrated that perceived sustainability value enhances tourists’ attitudes toward AR experiences, especially when AR is positioned as a tool for learning about and practicing sustainable tourism. These findings suggest that AR adoption is not merely a matter of technological appeal but also a reflection of the sustainability values perceived by users. Thus, we proposed the following hypothesis:

#### Hypothesis H5

AR adoption has a significant positive relationship with Perceived Sustainability Value.

### AR and innovation orientation

Innovation orientation is defined as the organizational or individual predisposition to embrace new ideas, experiment with novel solutions, and adopt cutting-edge technologies to create value^78^. In tourism, innovation orientation represents the willingness of firms and tourists alike to explore emerging technologies such as AR to enhance service delivery, improve customer experience, and differentiate offerings. Eco-smart tourism ventures, which integrate sustainability principles with technology-driven strategies^27^, rely heavily on innovation orientation to stay competitive and relevant in a market increasingly shaped by digital transformation and environmental responsibility.

Furthermore, innovation orientation contributes to cultivating a proactive mindset toward sustainability-driven technologies. From the tourists’ perspective, individuals with stronger innovation orientation are more likely to experiment with AR applications during their travel experiences. Research of Shin and Jeong^79^ shows that tourists who view themselves as adventurous and open to technological novelty perceive AR as a means to enrich their journey with educational and interactive content. However, rather than positioning innovation orientation as a precursor to AR adoption, it is more appropriate to conceptualize it as a response that is reinforced through direct interaction with AR enabled environments.

From a theoretical perspective grounded in the S-O-R framework, AR adoption is conceptualized as a technological stimulus that shapes tourists’ internal cognitive and behavioural tendencies. Within this framework, innovation orientation represents an organism level response, reflecting the extent to which exposure to AR technologies enhances tourists’ openness to innovation, experimentation, and technology driven experiences^80^. From a managerial perspective, innovation orientation can be viewed as a strategic outcome that eco smart tourism ventures aim to cultivate through the implementation of AR technologies. By exposing tourists to immersive and interactive digital environments, AR not only enhances immediate experiences but also fosters a longer-term orientation toward innovation and technology adoption^81^.

Empirical evidence reinforces the role of AR in shaping innovation related perceptions and behaviours. Gui et al.^82^ highlighted that interaction with advanced technologies strengthens users’ innovative thinking and willingness to experiment with new digital solutions. In the tourism sector, Elkhwesky et al.^83^ emphasized that digital technologies contribute to fostering innovative experiences by encouraging exploration and creativity among users. More recent studies by Jiang et al.^84^ demonstrated that AR applications enhance experiential novelty, which in turn stimulates users’ openness to innovative service encounters. Similarly, Alam et al.^21^ confirmed that engagement with AR based travel services enhances users’ receptiveness toward technology driven experiences. Accordingly, this study conceptualizes innovation orientation as a direct outcome of AR adoption in eco smart tourism ventures, reflecting tourists’ enhanced openness to innovation and technology driven experiences. Therefore, we propose the following hypothesis:

#### Hypothesis H6

AR adoption has a significant positive relationship with innovation orientation.

### AR and digital transformation of eco-smart tourism ventures

Digital transformation in tourism refers to the strategic integration of digital technologies into business models, processes, and customer experiences, resulting in enhanced competitiveness, sustainability, and innovation^85^. For eco-smart tourism ventures, digital transformation involves not only adopting technologies that improve operational efficiency but also leveraging them to foster sustainability, enrich tourist experiences, and build environmentally conscious business practices. AR plays a central role in facilitating digital transformation by enabling new forms of value co-creation between tourism providers and tourists^38^. Unlike static digital solutions, AR has ability to transforms eco-smart tourism ventures into dynamic platforms for experiential learning and engagement. In this study, AR adoption is conceptualized as a central independent and exogenous construct within the S-O-R framework, representing the technological stimulus introduced by eco-smart tourism ventures as a strategic decision. This implies that AR adoption is not treated as an outcome of psychological or perceptual factors, but rather as an organisational level initiative that shapes subsequent tourist experiences and responses. By positioning AR adoption as the stimulus, the model ensures conceptual clarity and alignment with S-O-R logic, where technological implementation precedes and influences internal and behavioural outcomes.

Additionally, AR enhances operational and marketing strategies within eco-smart tourism ventures, contributing to their broader digital transformation agenda. By integrating AR applications into mobile platforms, guided tours, and smart destinations, tourism providers can reduce dependence on traditional resources such as printed materials and physical signage, thus supporting green practices^48^. From the tourists’ perspective, AR adoption contributes to digital transformation by reshaping customer journeys and expectations. Tourists who engage with AR applications expect seamless, immersive, and tech-enabled experiences^56^, thereby pushing tourism providers to re-engineer their services around digital platforms. This demand-driven transformation reinforces a cycle where AR not only enhances tourist satisfaction and engagement but also accelerates the broader digitalization of eco-smart tourism ecosystems. In this way, AR adoption does not occur in isolation but becomes a pivotal element in the transformation of business models toward sustainability, innovation, and competitiveness. Within the S-O-R structure, digital transformation is conceptualized as the response, representing the organisational outcomes that emerge as a result of AR adoption and the subsequent organism level states such as immersive experience, environmental awareness support, cultural enrichment, trust, perceived sustainability value, innovation orientation, and tourist engagement. This positioning ensures that digital transformation is understood as an outcome influenced both directly by AR adoption and indirectly through tourist related experiential and psychological mechanisms.

Empirical studies underscore the transformative impact of AR on tourism. Jalilvand and Ghasemi^86^ found that AR applications significantly improve customer engagement and service personalization, which are core dimensions of digital transformation in hospitality and tourism. Similarly, Neuburger et al.^87^ highlighted that AR enhances destination marketing and tourist learning, facilitating a shift toward digitally immersive and sustainable practices. Moreover, research by Buhalis et al.^88^ revealed that AR adoption drives digital transformation by enabling smart tourism experiences and supporting sustainability agendas. Collectively, these findings suggest that AR is not merely a technological add-on but a strategic enabler of digital transformation in eco-smart tourism ventures. Therefore, we propose the following hypothesis:

#### Hypothesis H7

AR adoption has a significant positive relationship with the digital transformation of eco smart tourism ventures.

### Mediating role of tourist engagement

Tourist engagement has emerged as a critical construct in the tourism and hospitality literature, reflecting the depth of a tourist’s cognitive, emotional, and behavioural involvement in tourism experiences^89^. It is not limited to mere participation but encompasses active interaction, immersion, and co-creation of value in the tourism journey. Engagement serves as a bridge between technological adoption and broader outcomes such as loyalty, satisfaction, and transformation of business models. In the context of eco-smart tourism ventures, where sustainability and meaningful experiences are paramount, tourist engagement plays a pivotal mediating role in shaping how emerging technologies, particularly AR, translate into digital transformation outcomes.

Augmented Reality, with its immersive and interactive features, provides stimuli that spark curiosity, enhance enjoyment, and encourage learning among tourists^90^. However, the true value of AR lies not just in its adoption but in how it facilitates active engagement, prompting tourists to interact with both the digital environment and the physical eco-tourism setting. For instance, AR applications can allow visitors to visualize endangered ecosystems, experience local cultural narratives, or engage in interactive conservation tasks. According to Wen^91^, AR foster deeper engagement by stimulating cognitive involvement (knowledge acquisition), affective involvement (emotional connection), and behavioural involvement (active participation in sustainable practices). In this sense, AR becomes the stimulus that triggers engagement (the organism), which in turn drives behavioural outcomes such as supporting eco-tourism initiatives and fostering loyalty, aligning closely with the S-O-R framework.

Furthermore, engaged tourists are more likely to contribute to the digital transformation of eco-smart tourism ventures by acting as co-creators of value. Tourists active interactions with AR platforms produce valuable data, insights, and digital footprints that tourism operators can use to refine and innovate their offerings^92^. This aligns with the idea that tourist engagement is not only an individual-level outcome but also a collective resource that contributes to organizational transformation. For eco-smart ventures seeking to balance sustainability with competitiveness, engagement ensures that AR adoption translates into meaningful, eco-conscious digital transformation rather than mere technological novelty.

Empirical studies support the mediating role of engagement between technology adoption and transformational outcomes. For example, research in smart tourism contexts shows that AR adoption enhances tourist engagement, which subsequently leads to higher satisfaction, positive word-of-mouth, and sustainable destination loyalty^93^. Similarly, in eco-tourism, engagement has been found to mediate the relationship between experiential technologies and sustainable behavioural intentions^94^. These findings highlight that engagement is the key mechanism through which AR adoption yields long-term benefits for both tourists and eco-smart ventures. Based on this theoretical and empirical grounding, the following hypotheses are proposed:

#### Hypothesis H8

AR adoption has a significant positive relationship on tourist engagement.

#### Hypothesis H9

Tourist engagement has a significant positive relationship on the digital transformation of eco-smart tourism ventures.

#### Hypothesis H10

Tourist engagement has a significant mediating effect between AR adoption and the digital transformation of eco-smart tourism ventures.

### Conceptual framework

**Fig. 1:**
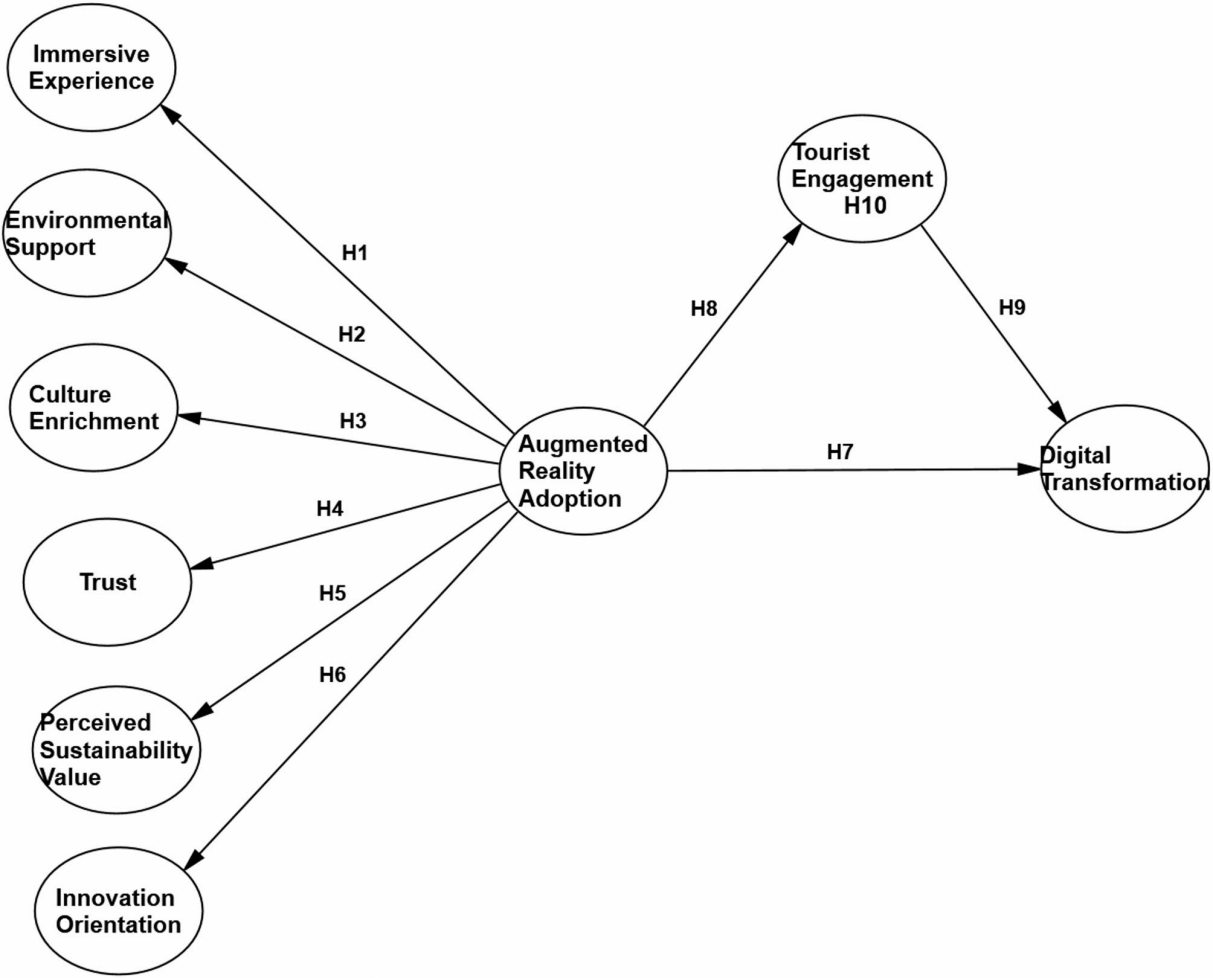
Conceptual framework.

Building upon the reviewed literature, this study proposes a conceptual framework that links Augmented Reality (AR) adoption, Tourist Engagement, and the Digital Transformation of eco-smart tourism ventures. The framework posits that AR adoption directly influences both Tourist Engagement and Digital Transformation, while Tourist Engagement itself exerts a positive influence on Digital Transformation. Moreover, Tourist Engagement is hypothesized to play a mediating role in the relationship between AR adoption and Digital Transformation, suggesting that the full potential of AR in eco-smart ventures is realized when tourists are actively engaged. This framework, grounded in the Stimulus–Organism–Response (S-O-R) perspective, highlights the transformative capacity of AR-enabled experiences to enhance engagement and drive sustainable digital innovation within eco-smart tourism. The proposed conceptual framework is illustrated in Fig. 1.

To strengthen theoretical consistency, this study explicitly positions all constructs within the S-O-R framework. In this context, AR adoption is conceptualized as the stimulus, representing the external technological input introduced by eco smart tourism ventures. This stimulus reflects the extent to which AR based applications and features are integrated into tourism offerings to enhance visitor experiences.

The organism component captures the internal cognitive and affective states of tourists that arise in response to AR adoption. Specifically, immersive experience, environmental awareness support, cultural enrichment, trust, perceived sustainability value, and innovation orientation are conceptualized as organism level responses rather than as independent drivers of AR adoption. These constructs reflect how tourists perceive, interpret, and emotionally respond to AR enabled experiences within eco smart tourism settings. By positioning these dimensions as organism states, the framework ensures alignment between the theoretical logic and the empirical model, where AR adoption is expected to shape these perceptions and experiential outcomes.

Furthermore, Tourist Engagement is positioned as a higher order organism construct that represents the behavioural and psychological manifestation of these internal states. In other words, the six organism level dimensions collectively contribute to fostering deeper tourist engagement, which reflects active participation, emotional connection, and cognitive involvement with the tourism experience. This hierarchical conceptualization strengthens the explanatory power of the organism component within the S-O-R framework.

The response component is represented by the Digital Transformation of eco smart tourism ventures, which captures organisational level outcomes associated with the adoption of AR technologies. In this study, digital transformation is reflected through improvements in innovation practices, service delivery, and sustainable business performance. The framework therefore proposes that AR adoption influences digital transformation both directly and indirectly through its impact on organism level states and subsequent tourist engagement.

Importantly, this conceptualization resolves prior ambiguity in the literature regarding the role of AR related dimensions by clearly defining them as outcomes of AR adoption at the organism level. This ensures consistency between construct definitions, measurement, and model specification. Accordingly, the framework advances the S-O-R theory by integrating technology driven stimuli with multi-dimensional organism responses and linking them to firm level transformation outcomes in the context of eco smart tourism ventures. This provides a more nuanced and theoretically grounded explanation of how AR adoption is associated with digital transformation through experiential and engagement-based mechanisms.

## Methodology

### Research design

The study adopts a quantitative research design grounded in the positivist paradigm, which emphasizes objectivity, measurement, and the testing of hypotheses through empirical data^95^. The positivist stance assumes that reality is observable, measurable, and independent of the researcher, making it appropriate for investigating the influence of AR adoption on the digital transformation of eco-smart tourism ventures. Within this paradigm, knowledge is generated through systematic collection and statistical analysis of data to identify causal relationships among variables.

The quantitative approach is selected because it allows for the testing of well-defined hypotheses derived from the literature review and conceptual framework. Specifically, this study seeks to examine the direct effects of cultural enrichment, trust, perceived sustainability value, and innovation orientation on AR adoption, as well as the mediating role of tourist engagement in the relationship between AR adoption and digital transformation of eco-smart tourism ventures. Quantitative methods enable the measurement of constructs through structured survey instruments, providing standardized and reliable data suitable for inferential analysis^96^.

Through employing this research design, the study is able to generalize findings across eco-smart tourism ventures and establish statistically valid conclusions. The use of structural equation modelling (SEM) is proposed for data analysis, as it facilitates testing both direct and indirect relationships among the constructs in the conceptual framework^97^. This rigorous methodological approach ensures that the study not only contributes theoretically to the literature on AR adoption and eco-smart tourism but also provides practical implications for entrepreneurs, policymakers, and stakeholders in the tourism industry seeking to harness digital transformation for sustainability.

### Questionnaire design

The primary data for this study will be collected using a structured questionnaire, which serves as the most suitable tool for gathering standardized responses across a large number of participants. The questionnaire is developed based on validated measurement items from prior studies, adapted to the context of AR adoption, tourist engagement, and the digital transformation of eco-smart tourism ventures. To ensure clarity and relevance, the questionnaire is organized into distinct sections that correspond to the main constructs of the conceptual framework.

The first section consists of demographic information, capturing variables such as age, gender, education, occupation, travel experience, and frequency of participation in eco-tourism activities. These variables provide background information that may be useful for subgroup analysis and understanding respondent characteristics. The second section includes measurement items related to the independent variables, such as cultural enrichment, trust, perceived sustainability value, and innovation orientation, which are conceptualized as organism level responses influenced by AR adoption. The third section focuses on the mediating construct, tourist engagement, which assesses how visitors interact, participate, and develop emotional or cognitive connections through AR experiences. The fourth section measures the dependent variable, namely the digital transformation of eco-smart tourism ventures, which reflects how AR facilitates innovation, technological integration, and sustainable value creation in tourism enterprises.

All items are measured using a 7-point Likert scale, ranging from “1 = Strongly Disagree” to “7 = Strongly Agree.” This scale is chosen because it provides greater sensitivity and variance in responses, allowing for more accurate statistical analysis. The use of Likert scaling ensures that respondents can express their level of agreement or disagreement with each statement, enabling the study to capture perceptions and attitudes in a quantitative form^98^.

Since the study is conducted in Malaysia, the questionnaire is designed in English and translated into Bahasa Malaysia to ensure better comprehension among respondents. The translation process follows the back-translation method to maintain semantic and conceptual equivalence between both versions. This approach reduces the risk of misinterpretation and enhances the reliability and validity of the responses.

The study adopts a cross-sectional survey design, where data will be collected at a single point in time. This design is appropriate for testing relationships between variables in the conceptual framework and provides insights into how AR adoption contributes to eco-smart tourism transformation through tourist engagement. The structured nature of the questionnaire, combined with the use of Likert-scale items, ensures that the collected data is suitable for advanced statistical techniques.

Given that all constructs were measured using self-reported data collected from the same respondents at a single point in time, the potential for common method bias was carefully considered and addressed through procedural remedies. Procedurally, several steps were taken to minimize response bias. First, respondents were assured of anonymity and confidentiality to reduce evaluation apprehension and social desirability bias. Second, the questionnaire items were carefully structured with clear and simple wording to avoid ambiguity and reduce the likelihood of common scale effects. Third, the measurement items for different constructs were organized into separate sections to create psychological separation and reduce respondents’ tendency to provide consistent answers across variables.

### Measurement of variables

To empirically validate the proposed conceptual framework, this study employs established constructs adapted from prior literature. Each construct is measured using multiple reflective indicators to ensure internal consistency, validity, and comparability with previous studies. Below, the key terms are defined and linked to their corresponding measurement items and sources.

*Immersive experience* refers to the extent to which tourists perceive a sense of presence, interactivity, and deep engagement through AR technologies, enabling them to feel actively involved in the tourism activity. Measurement items were adapted from Carù and Cova^99^, Tussyadiah et al.^100^, and Rauschnabel et al.^101^.

*Environmental awareness support* captures the degree to which AR enhances tourists’ awareness, learning, and appreciation of sustainability, ecological protection, and environmental practices during eco-tourism experiences. Measurement items were adapted from Kiatkawsin and Han^102^, and Chiu et al.^103^.

*Trust* refers to tourists’ belief that AR technology and eco-tourism providers are dependable, secure, and capable of delivering accurate and reliable information that improves the quality of their experience. Measurement items were adapted from Razak et al.^104^ and Su et al.^105^.

*Perceived sustainability value* reflects tourists’ perceptions that AR adoption contributes to environmental preservation, cultural continuity, and social sustainability, thereby creating long-term value in eco-tourism ventures. Measurement items were adapted from Samaddar and Mondal^48^ and Wismantoro et al.^106^.

*Innovation orientation* refers to the degree to which eco-tourism ventures strategically adopt AR technologies to innovate services, enhance competitiveness, and create differentiated tourism experiences. Measurement items were adapted from Alam et al.^21^ and Tang et al.^107^.

*Tourist engagement* represents the cognitive, emotional, and behavioural involvement exhibited by tourists during AR-enhanced eco-tourism experiences, which strengthens their interaction and satisfaction. Measurement items were adapted from tom Dieck et al.^108^, and Dağ et al.^39^.

*Digital transformation of eco-smart tourism ventures* refers to the integration of AR and other digital technologies into eco-tourism operations, thereby enhancing sustainability practices, operational efficiency, and visitor experiences. Measurement items were adapted from Avilés-Sacoto et al.^109^ and Khalil et al.^110^.

### Pilot test

Prior to conducting the main survey, a pilot test was carried out to ensure the reliability, clarity, and appropriateness of the research instrument. The pilot study served as a preliminary assessment to refine the questionnaire, identify potential ambiguities, and confirm that the constructs and items were well understood by the target respondents. Following the recommendations of Johanson and Brooks^111^, a sample size of 30 respondents was considered adequate for pilot testing, as this number is generally sufficient to detect errors and test the consistency of items. The pilot participants were selected from individuals with prior eco-tourism experience and familiarity with technology-based tourism applications, making them representative of the actual study population.

The pilot test specifically evaluated the content validity, face validity, and reliability of the measurement scales^112^. Content and face validity were examined by consulting academic experts in tourism and digital technology, as well as by gathering feedback from respondents regarding item clarity and language comprehension. Since the questionnaire was originally developed in English and subsequently translated into Bahasa Malaysia to enhance understanding among local respondents, the pilot also tested the accuracy of translation and cultural appropriateness of the terms used.

Reliability was assessed using Cronbach’s alpha for each construct, with results exceeding the recommended threshold of 0.70^113^, indicating acceptable internal consistency. Based on the feedback received, minor revisions were made to wording for clarity, but no items were removed. Overall, the pilot test confirmed that the questionnaire was reliable, valid, and well-suited for the main study. The results demonstrated that all items and constructs achieved satisfactory levels of significance, thereby allowing the research to confidently proceed with the full-scale data collection.

### Sampling and procedure

In Malaysia, small tourism ventures are typically defined according to the SME Corporation Malaysia classification, which categorizes service-based SMEs by the number of employees and annual sales turnover. Specifically, small ventures in the tourism and hospitality sector are characterized by having 5 to 30 full-time employees and an annual turnover of RM300,000 to RM3 million. These businesses play a crucial role in the eco-tourism ecosystem, offering niche services such as eco-lodges, cultural experiences, heritage tours, and technology-enhanced tourism products. Such ventures represent the target population for this study as they are key stakeholders in the adoption of AR and digital transformation within eco-smart tourism practices.

The sampling population of this study consisted of owners, managers, and senior employees of small tourism ventures across Malaysia. Given the geographical diversity of eco-tourism operations in the country, the study focused on seven states with high tourism activity and eco-tourism potential: Sarawak, Sabah, Penang, Johor, Selangor, Melaka, and Pahang. These states were selected as they represent different eco-tourism environments, from natural parks and heritage sites to urban tourism hubs.

For determining the appropriate sample size, this study referred to the widely recognized sample size determination table by Krejcie and Morgan^114^, which provides guidance at a 95% confidence level with a 5% margin of error. Given that the population of small tourism ventures in Malaysia is estimated to exceed 10,000 (SME Corp Malaysia, 2023), the table recommends a minimum sample size of 384 respondents. To ensure adequate representation across the seven states and to mitigate potential non-responses or incomplete questionnaires, the study targeted 400 respondents. Ultimately, 367 valid questionnaires were collected, representing a response rate of 91.75%. This sample size is deemed sufficient for further analysis, as Hair et al.^115^ emphasized that a minimum of 200 responses is considered the threshold for structural equation modelling (SEM).

The sampling technique adopted was a stratified random sampling approach, where respondents were proportionately drawn from each of the selected states to ensure adequate representation. Specifically, proportional allocation was implemented based on the estimated distribution of small tourism enterprises across the selected states, using available data from SME Corp Malaysia and state level tourism directories as a proxy for SME density. This approach ensured that states with a higher concentration of tourism related SMEs contributed a correspondingly larger share of respondents, while states with fewer enterprises were proportionately represented, thereby improving the representativeness of the sample. Within each state, businesses were identified through local tourism boards, SME directories, and associations, after which owners, managers, and senior employees were invited to participate. This stratification procedure enhances the generalizability of the findings by reducing sampling bias and ensuring that the diversity of eco smart tourism ventures across different regional contexts in Malaysia is adequately captured.

The data collection procedure was carried out through two methods: (i) self-administered surveys, where hard copies of questionnaires were distributed directly to respondents during visits to tourism sites and business premises; and (ii) online surveys, where a digital version of the questionnaire (via Google Forms) was disseminated through email, WhatsApp, and professional tourism networks. This hybrid approach was employed to increase the response rate and overcome geographical limitations, especially for remote states such as Sabah and Sarawak. Respondents were assured of confidentiality and anonymity, and participation was entirely voluntary. Data collection spanned a period of three months to ensure adequate coverage and response distribution across the selected states.

### Statistical analysis

The data collected from small tourism ventures across seven states in Malaysia were analysed using a combination of descriptive, inferential, and SEM techniques. The analysis process was designed to provide both preliminary insights and robust testing of the proposed research hypotheses.

Firstly, the data were screened and coded using Statistical Package for Social Sciences (SPSS) version 28.0. SPSS was employed to conduct descriptive statistics, including means, standard deviations, normality assessment, reliability tests were conducted at this stage to ensure internal consistency of the measurement items. Subsequently, hypothesis testing was carried out using Analysis of Moment Structures (AMOS) version 24.0, applying the SEM approach. The SEM analysis followed the widely recognized two-stage process.

In the first stage, the measurement model was assessed to establish the unidimensionality of the constructs. This involved performing Confirmatory Factor Analysis (CFA) to evaluate the factor loadings of the items, as well as testing for model fit using indices such as the Comparative Fit Index (CFI), Tucker-Lewis Index (TLI), Root Mean Square Error of Approximation (RMSEA), and Chi-square/df ratio. Convergent validity and discriminant validity were also examined. In the second stage, the structural model was tested to evaluate the hypothesized relationships between constructs. This stage focused on examining the path coefficients and their significance levels, thereby confirming or rejecting the proposed hypotheses. To further test the mediating role of tourist engagement, the bootstrapping method was employed.

## Findings

### Demographic findings

The demographic profile of the respondents provides meaningful insights into the characteristics of small tourism ventures in Malaysia (Table 1). In terms of gender distribution, male respondents (54.2%) slightly outnumber female respondents (40.1%), while a small proportion (5.7%) preferred not to disclose their gender. This suggests a fairly balanced representation, indicating that both male and female stakeholders are actively involved in eco-smart tourism ventures.

With respect to age, the largest group of respondents falls within the 35–44 years category (32.4%), followed by those aged 45–54 years (22.6%). This reflects that tourism ventures are largely managed by individuals in their mid-career stage, who are likely to have accumulated substantial professional and managerial experience. Interestingly, younger respondents below 25 years represent only 9.3%, signalling that while youth engagement in eco-smart tourism is emerging, it remains relatively limited.

In terms of organizational roles, nearly half of the respondents identified themselves as owners or founders (42%), followed by managers/administrators (24.2%) and co-owners/partners (18.2%). This indicates that the dataset is largely representative of decision-makers and key influencers within the ventures, which strengthens the reliability of responses regarding strategic adoption of technologies such as Augmented Reality (AR).

Educational qualifications highlight that a majority of respondents possess higher education, with 41.4% holding a bachelor’s degree and 22.3% a master’s degree. This reflects a relatively educated workforce within eco-tourism ventures, which may facilitate openness to technological innovations and sustainability practices.

Regarding the type of tourism ventures, travel and tour agencies (26.7%) and eco-lodges/resorts (25.3%) dominate the sample, followed by food and beverage establishments (18.8%) and adventure/outdoor activity providers (13.9%). This distribution underscores the diversity of eco-smart tourism models in Malaysia, spanning accommodation, guided experiences, cultural attractions, and eco-themed consumption.

In terms of business size, the majority of ventures fall within the micro and small enterprise categories, with 33.5% employing 5–10 full-time staff and 25.6% employing 11–30 staff. This confirms that the sector is dominated by lean organizations, aligning with the SME characteristics emphasized by SME Corp Malaysia. Similarly, most ventures are relatively young, with 36% operating for 4–6 years and 25.6% for 7–10 years, pointing toward a growing eco-tourism industry driven by relatively new entrants.

Geographical distribution shows the highest concentration of ventures in Sarawak (26.4%), followed by Johor (17.7%) and Selangor (13.9%), reflecting the strong presence of eco-tourism hubs in both East and Peninsular Malaysia.

Digital readiness findings reveal that the majority of ventures operate at a moderate level of digital adoption (44.4%), integrating tools such as online booking systems and e-payments, while only 6.8% reported very high adoption of advanced technologies such as AR, VR, or AI. This highlights the gradual digital transition of the sector. Finally, in terms of AR adoption, the majority of ventures are either aware but not yet adopted (36%) or planning to adopt soon (31.9%), with only 4.6% reporting full integration into core operations. This finding underscores the emerging but still nascent role of AR in transforming eco-smart tourism ventures in Malaysia.

In particular, these demographic findings reveal that eco-smart tourism ventures are largely owner-driven, moderately digitalized, and concentrated in growth-oriented states, with an encouraging awareness of AR technologies, yet significant opportunities remain for scaling adoption to drive digital transformation.

**Table 1:** Demographic findings.

Characteristics		Number	Percentage
Gender	Male	199	54.2
	Female	147	40.1
	Prefer not to say	21	5.7
Age group	Below 25 years	34	9.3
	25–34 years	73	19.9
	35–44 years	119	32.4
	45–54 years	83	22.6
	55 years and above	58	15.8
Position in the business	Owner/Founder	154	42.0
	Co-owner/Partner	67	18.2
	Manager/Administrator	89	24.2
	Marketing/Operations staff	38	10.4
	Other	19	5.2
Education qualification	Secondary school (SPM or equivalent)	31	8.4
	Diploma/Certificate	79	21.5
	Bachelor’s degree	152	41.4
	Master’s degree	82	22.3
	Doctorate (PhD/DBA)	12	3.3
	Other	11	3.1
Type of tourism venture	Eco-lodge/Guesthouse/Resort	93	25.3
	Travel and Tour Agency	98	26.7
	Adventure/Outdoor Activities Provider (e.g., hiking, diving, cycling)	51	13.9
	Eco-tourism Attraction (parks, cultural villages, heritage sites)	7	1.9
	Food & Beverage (eco-themed cafes/restaurants)	69	18.8
	Handicraft/Local Products Enterprise	32	8.7
	Other	17	4.7
Business size (No. of full-time employees)	Less than 5 employees (micro)	61	16.6
	5–10 employees	123	33.5
	11–30 employees	94	25.6
	31–50 employees	59	16.1
	51–75 employees	30	8.2
Years of business operation	Less than 1 year	21	5.7
	1–3 years	82	22.3
	4–6 years	132	36.0
	7–10 years	94	25.6
	More than 10 years	38	10.4
Geographical location of the business	Sarawak	97	26.4
	Sabah	38	10.4
	Johor	65	17.7
	Selangor	51	13.9
	Penang	43	11.7
	Melaka	46	12.5
	Pahang	27	7.4
Level of digital technology adoption in business operations	Very low (minimal use of digital tools)	16	4.4
	Low (basic use – social media, website)	91	24.8
	Moderate (online booking, e-payments)	163	44.4
	High (integrated digital marketing, analytics)	72	19.6
	Very high (advanced use including AR/VR/AI tools)	25	6.8
Current status of Augmented Reality (AR) adoption in your business	Not aware of AR technologies	29	7.9
	Aware but not yet adopted	132	36.0
	Planning to adopt soon	117	31.9
	Adopted partially (trial/limited functions)	72	19.6
	Fully adopted in core operations	17	4.6

### Normality findings

To ensure the suitability of the data for further statistical analyses, both univariate and multivariate normality were assessed. As shown in Table 2, the univariate normality test results indicate that the skewness and kurtosis values for all constructs fall within the acceptable range of − 2 to + 2, as recommended by Cain et al.^116^. This suggests that the data distribution for each construct, including immersive experience, environmental awareness support, cultural enrichment, trust, perceived sustainability value, innovation orientation, tourist engagement, and digital transformation, meets the criteria for univariate normality. Specifically, skewness values range from − 0.062 to 0.188, while kurtosis values range from − 0.079 to 0.873, reflecting a balanced distribution with no severe deviations from normality.

In terms of multivariate normality, the critical ratio for multivariate skewness and kurtosis was found to be 0.382 and 0.567 respectively, both of which fall within acceptable limits, further confirming that the dataset does not suffer from multivariate non-normality. This is particularly important given the application of SEM, where assumptions of normality are critical for robust parameter estimation and valid hypothesis testing.

**Table 2:** Normality findings.

Variables	Mean	Std. Dev.	Skewness	c.*r*.	Kurtosis	c.*r*.
Immersive experience	5.44	0.43	0.103	0.942	− 0.052	− 0.387
Environmental awareness support	5.76	0.47	0.077	0.674	0.269	1.734
Cultural enrichment	6.25	0.36	0.158	1.693	0.088	0.494
Trust	5.18	0.41	0.084	0.781	0.091	0.529
Perceived sustainability value	5.87	0.39	0.039	0.047	0.446	0.905
Innovation orientation	5.92	0.49	− 0.062	− 0.562	0.032	0.072
Tourist engagement	6.18	0.56	0.188	1.95	0.873	1.231
Digital transformation	5.62	0.45	0.047	0.533	− 0.079	− 0.478
Multivariate					0.382	0.567

### Findings of discriminant validity

Discriminant validity was assessed using the Heterotrait-Monotrait (HTMT) ratio of correlations, which is considered a more reliable criterion than the Fornell-Larcker approach for evaluating construct distinctiveness^117^. As shown in Table 3, all HTMT values across the constructs fall well below the conservative threshold of 0.85 and the more liberal threshold of 0.90, indicating that each construct is empirically distinct from the others. For instance, the HTMT value between immersive experience and tourist engagement is 0.56, while the correlation between cultural enrichment and innovation orientation is 0.53, both comfortably within acceptable limits. Similarly, the relationships between trust and environmental awareness support (0.46), as well as between perceived sustainability value and digital transformation (0.48), demonstrate adequate discriminant separation, ensuring that overlapping variance does not undermine the uniqueness of each construct.

**Table 3:** HTMT findings.

Variables	1	2	3	4	5	6	7	8
Immersive experience	1							
Environmental awareness support	0.39	1						
Cultural enrichment	0.45	0.37	1					
Trust	0.42	0.46	0.34	1				
Perceived sustainability value	0.32	0.41	0.48	0.43	1			
Innovation orientation	0.35	0.47	0.53	0.44	0.36	1		
Tourist engagement	0.56	0.33	0.49	0.31	0.54	0.38	1	
Digital transformation	0.46	0.42	0.36	0.37	0.48	0.32	0.58	1

### Findings of confirmatory factor analysis (CFA)

**Fig. 2:**
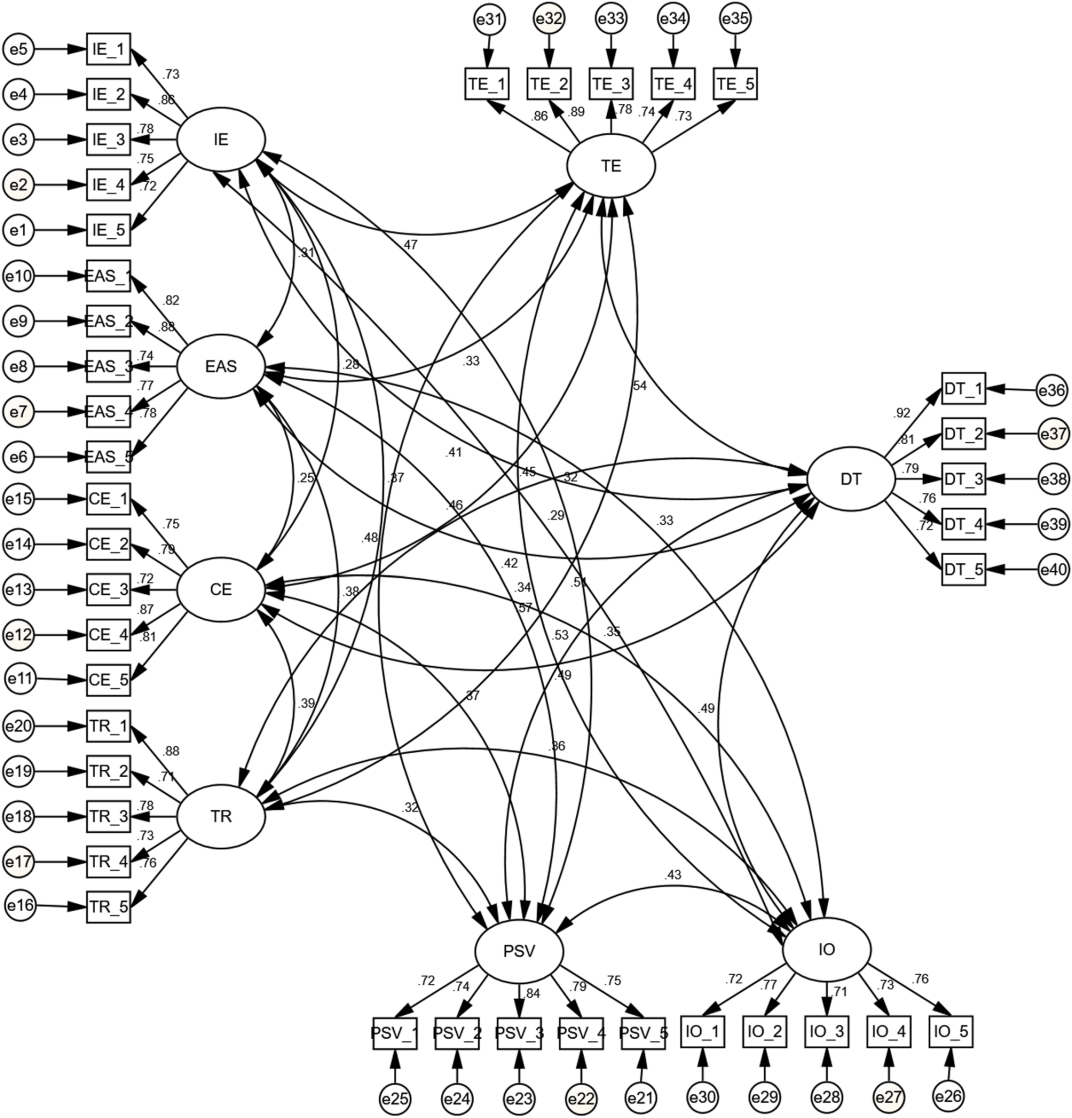
Overall measurement model. *Note*: IE (Immersive experience), EAS (Environmental awareness support), CE (Cultural enrichment), TR (Trust), PSV (Perceived sustainability value), IO (Innovation orientation), TE (Tourist engagement) and DT (Digital transformation).

To validate the measurement model, Confirmatory Factor Analysis (CFA) was conducted to assess the unidimensionality, reliability, and validity of the constructs. CFA provides a rigorous test of how well the observed variables represent their underlying latent constructs and ensures that the measurement items align with theoretical expectations^118^. The analysis was carried out using AMOS, and the results indicated a robust model fit across multiple indices. As illustrated in Fig. 2, the model fit indices met the recommended thresholds: RMSEA = 0.031, Chi-square (χ²) = 374.982 with df = 366, GFI = 0.915, AGFI = 0.931, CFI = 0.948, and CMIN/df = 3.314. It is important to note that the reported AGFI value appears slightly higher than the GFI value, although AGFI is typically expected to be lower than GFI. However, such instances may occur due to estimation procedures and sample characteristics in covariance-based SEM^119^. Moreover, the CMIN/df value of 3.314 falls within the acceptable range rather than indicating a perfect fit. Dash and Paul^119^ suggest that values below 3, or in some cases below 5, may be considered acceptable depending on model complexity and sample size. Therefore, the overall model fit can be interpreted as adequate to good, reflecting a well-fitting measurement model that is consistent with established SEM evaluation criteria.

Beyond model fit, the Average Variance Extracted (AVE) and Composite Reliability (CR) values were examined to assess convergent validity and internal consistency^113^. As presented in Table 4, the AVE values for all constructs exceeded the minimum threshold of 0.50, indicating that each construct explains more than half of the variance of its indicators. Similarly, all CR values were above 0.70, confirming strong reliability and internal consistency across the constructs. For example, constructs such as immersive experience, tourist engagement, and digital transformation recorded AVE and CR values well above the thresholds, reflecting robustness in capturing the intended dimensions.

Although some factor loadings were moderately above the acceptable threshold, ranging between 0.71 and 0.72, these values are considered acceptable in CFA, particularly when supported by strong composite reliability and AVE values^120^. The retention of these items is theoretically justified, as they contribute meaningfully to the content validity of the constructs and do not adversely affect overall model fit. During the CFA refinement process, two measurement items from the innovation orientation and cultural enrichment constructs were removed due to relatively low factor loadings and high modification indices, which indicated potential redundancy and overlap with other items. The deletion of these items resulted in improved model fit and enhanced construct validity, while maintaining the conceptual integrity of the innovation orientation and cultural enrichment. This item purification process follows established SEM practices, where items are iteratively assessed and removed only when both statistical and theoretical justifications are met^121^. Importantly, apart from these refinements, no further item deletions were required, and all remaining items demonstrated satisfactory loadings, supporting the stability and robustness of the measurement model.

**Table 4:** CR and AVE findings.

Variables	Items	Measurement path	Factor loading
Immersive experience	IE_1	AR applications allow our visitors to feel fully engaged in the tourism experience.	0.73
	IE_2	AR makes our visitors feel as though they are part of the real environment.	0.86
	IE_3	Our visitors experience a high level of interactivity when using AR in tourism services.	0.78
	IE_4	AR provides our visitors with a memorable and stimulating experience.	0.75
	IE_5	The immersive experience of AR enhances visitors’ enjoyment during eco-tourism activities.	0.72
	CR		0.879
	AVE		0.592
Environmental awareness support	EAS_1	AR applications help our visitors better understand environmental issues.	0.82
	EAS_2	AR increases our visitors’ awareness of sustainable tourism practices.	0.88
	EAS_3	Using AR encourages our visitors to support eco-friendly behaviors.	0.74
	EAS_4	AR enhances our visitors’ knowledge of environmental conservation.	0.77
	EAS_5	AR motivates our visitors to value natural resources more responsibly.	0.78
	CR		0.898
	AVE		0.639
Cultural enrichment	CE_1	AR applications provide our visitors with rich cultural knowledge.	0.75
	CE_2	AR enhances our visitors’ understanding of local traditions and heritage.	0.79
	CE_3	Our visitors feel more connected to local culture through AR.	0.72
	CE_4	AR increases our visitors’ appreciation of cultural diversity in eco-tourism.	0.87
	CE_5	AR makes cultural learning more interesting and enjoyable for our visitors.	0.81
	CR		0.892
	AVE		0.624
Trust	TR_1	Our visitors trust the information provided through AR applications in tourism services.	0.88
	TR_2	AR technology is perceived as reliable for delivering accurate content to our visitors.	0.71
	TR_3	Our visitors feel safe using AR applications during their tourism experience.	0.78
	TR_4	Our visitors believe AR providers act in the best interest of tourists.	0.73
	TR_5	AR applications increase our visitors’ confidence in tourism services.	0.76
	CR		0.882
	AVE		0.599
Perceived sustainability value	PSV_1	AR creates value by supporting eco-friendly tourism experiences for our visitors.	0.72
	PSV_2	AR contributes to the sustainability of our tourism activities.	0.74
	PSV_3	Using AR benefits both our visitors and the environment.	0.84
	PSV_4	AR helps preserve cultural and natural resources in our tourism offerings.	0.79
	PSV_5	AR adoption is valuable for achieving sustainable tourism goals in our venture.	0.75
	CR		0.878
	AVE		0.592
Innovation orientation	IO_1	AR adoption reflects the innovative mindset of our tourism venture.	0.72
	IO_2	Our tourism venture uses AR to deliver creative and unique experiences.	0.77
	IO_3	Innovation is central to the use of AR in our eco-tourism services.	0.71
	IO_4	AR encourages the development of novel tourism practices within our venture.	0.73
	IO_5	We believe AR represents a future oriented innovation in eco-tourism.	0.76
	CR		0.857
	AVE		0.545
Tourist engagement	TE_1	Our visitors feel emotionally connected to AR based tourism experiences.	0.86
	TE_2	AR applications keep our visitors actively involved during their visit.	0.89
	TE_3	Our visitors are willing to spend time exploring AR enabled tourism experiences.	0.78
	TE_4	Our visitors feel enthusiastic about participating in AR based tourism activities.	0.74
	TE_5	AR applications make our visitors more engaged with eco-tourism.	0.73
	CR		0.900
	AVE		0.644
Digital transformation	DT_1	AR adoption supports the digital transformation of our eco-tourism venture.	0.92
	DT_2	AR enhances operational efficiency in our eco-tourism services.	0.81
	DT_3	AR enables the integration of sustainability into our digital strategies.	0.79
	DT_4	AR contributes to innovative and eco-smart business models in our tourism venture.	0.76
	DT_5	Our tourism venture becomes more competitive through AR driven transformation.	0.72
	CR		0.900
	AVE		0.645

### Findings of structural model

**Fig. 3:**
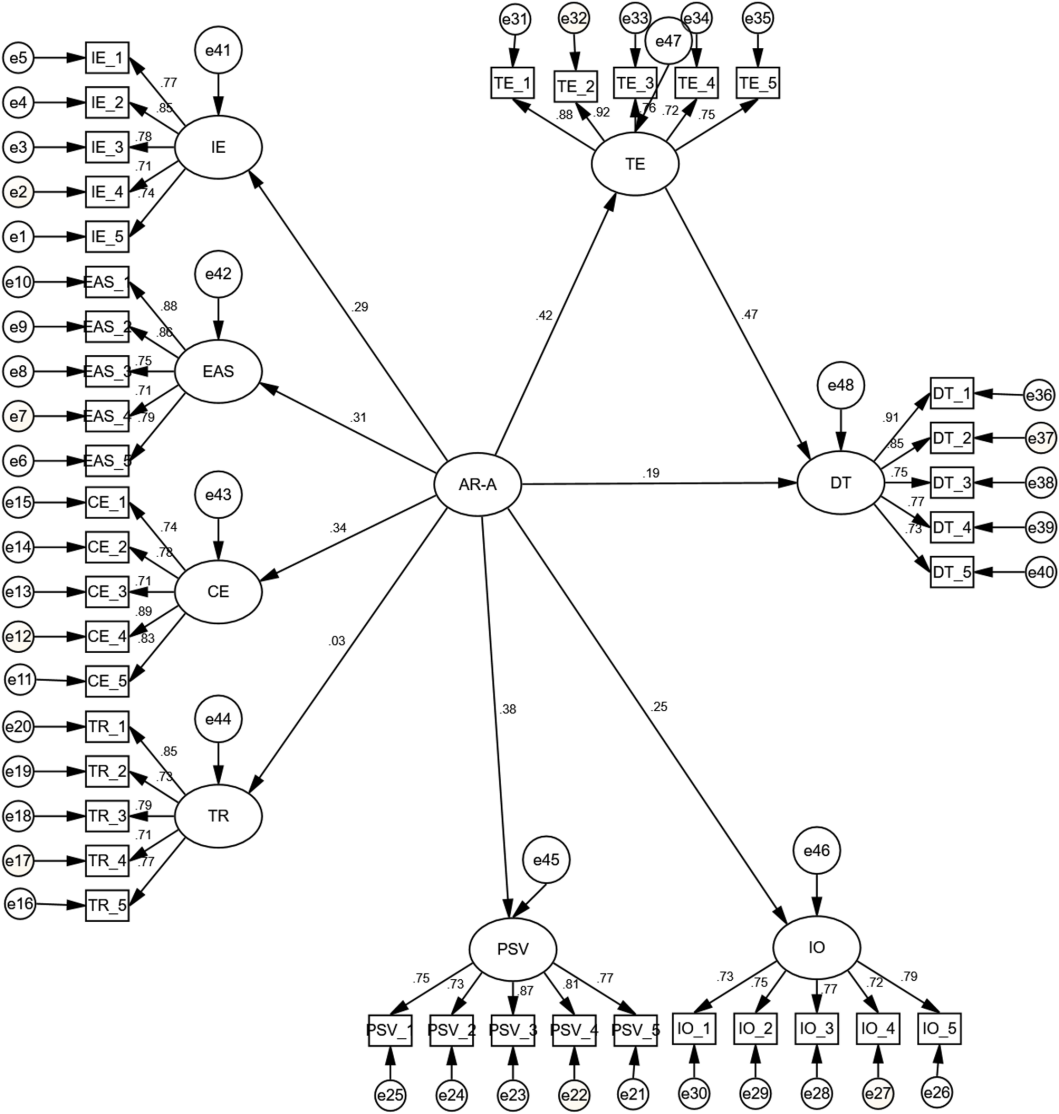
Structural model.

The structural model was assessed to test the hypothesized relationships^122^, and the findings are illustrated in Fig. 3. The model demonstrated a strong overall fit, with indices exceeding recommended thresholds (GFI = 0.951, AGFI = 0.933, CFI = 0.957, TLI = 0.945, RMSEA = 0.049, χ²/df = 3.913). These values indicate that the proposed structural model is robust and provides an excellent representation of the data, thereby enabling reliable interpretation of the hypothesized paths.

The findings, as summarized in Table 5, confirmed that augmented reality adoption (AR-A) significantly influenced most of the proposed dimensions of eco-smart tourism ventures. Specifically, Immersive Experience (IE) (β = 0.29, *p* < .001, z = 3.791, f² = 0.132), Environmental Awareness Support (EAS) (β = 0.31, *p* < .001, z = 3.904, f² = 0.163), and Cultural Enrichment (CE) (β = 0.34, *p* < .001, z = 4.227, f² = 0.179) were all positively associated with AR adoption. Similarly, Perceived Sustainability Value (PSV) emerged as the strongest outcome (β = 0.38, *p* < .001, z = 4.536, f² = 0.186), followed by Innovation Orientation (IO) (β = 0.25, *p* = .001, z = 3.468, f² = 0.117). These findings suggest that AR technologies are powerful enablers in creating enriched, sustainable, and innovative tourism experiences.

Interestingly, the relationship between AR adoption and Trust (TR) was not significant (β = 0.03, *p* = .182, z = 1.135, f² = 0.009). This indicates that, despite AR’s ability to provide immersive and engaging experiences, it does not automatically translate into increased trust from tourists. This finding is theoretically meaningful, suggesting that trust is not solely driven by technological stimuli such as AR, but rather depends on broader contextual factors such as service reliability, organisational reputation, credibility of information, and the specific trust target, whether in the technology or the tourism provider. In line with the S-O-R perspective, this result implies that trust may require additional reinforcing mechanisms beyond AR adoption, highlighting that eco-smart tourism ventures must complement AR implementation with trust building strategies such as transparent communication and consistent service quality.

This indicates that, despite AR’s ability to provide immersive and engaging experiences, it does not automatically translate into increased trust from tourists. This finding aligns with prior research suggesting that trust in tourism ventures often requires consistent service quality, reputation, and relational transparency beyond technological augmentation.

At the higher level, AR adoption demonstrated a direct positive influence on Digital Transformation (DT) (β = 0.19, *p* = .003, z = 3.051, f² = 0.078), underscoring its role as a catalyst for technological advancement in eco-smart tourism ventures. More importantly, AR adoption strongly enhanced Tourist Engagement (TE) (β = 0.42, *p* < .001, z = 4.878, f² = 0.189), which in turn exhibited a robust positive effect on digital transformation (β = 0.47, *p* < .001, z = 4.942, f² = 0.195). These results highlight that while AR adoption has a direct impact on transformation, its indirect influence, channelled through higher tourist engagement, is even more powerful, reinforcing the mediating role of engagement in driving digital transformation.

**Table 5:** Findings of structural model.

Hypothesis	β	*p*-value	z-value	f^2^	Findings
*H1*: IE <-- AR-A	0.29	0.000	3.791	0.132	Accepted
*H2*: EAS <-- AR-A	0.31	0.000	3.904	0.163	Accepted
*H3*: CE **<--** AR-A	0.34	0.000	4.227	0.179	Accepted
*H4*: TR <-- AR-A	0.03	0.182	1.135	0.009	Not accepted
*H5*: PSV <-- AR-A	0.38	0.000	4.536	0.186	Accepted
*H6*: IO <-- AR-A	0.25	0.001	3.468	0.117	Accepted
*H7*: AR-A --> DT	0.19	0.003	3.051	0.078	Accepted
*H8*: AR-A -->TE	0.42	0.000	4.878	0.189	Accepted
*H9*: TE --> DT	0.47	0.000	4.942	0.195	Accepted

AR-A (Augmented reality adoption).

As shown in Table 5, the f² thresholds of 0.02, 0.15, and 0.35 represent small, medium, and large effect sizes, respectively, as recommended by Cohen^123^.

### Findings of mediating effect

To further assess the mediating role of Tourist Engagement (TE), both direct and indirect pathways were examined. The results reveal that the direct effect of Augmented Reality adoption (AR-A) on the Digital Transformation (DT) of eco-smart tourism ventures was positive and statistically significant, supporting the hypothesized direct influence. At the same time, the indirect effect through Tourist Engagement was also found to be significant, demonstrating the presence of a complementary mediation effect. This indicates that while AR adoption independently contributes to digital transformation, its influence is amplified when tourists are actively engaged in AR-enhanced experiences.

A bootstrap resampling procedure with 1000 iterations at a 95% confidence interval confirmed the robustness of the findings. Standardized estimates as shown in Table 6, the pathway from AR-A to TE was strong and highly significant (β = 0.42, *p* < .001, z = 4.878), while the subsequent pathway from TE to DT was equally robust (β = 0.47, *p* < .001, z = 4.942). Importantly, the indirect effect of AR adoption on digital transformation through tourist engagement was also significant (β = 0.20, 95% CI = 0.14, 0.29), as the confidence interval does not include zero, thereby confirming the presence of a mediation effect. Together, these results confirm that Tourist Engagement serves as a powerful mechanism (complementary mediation) that channels the benefits of AR adoption into meaningful digital transformation outcomes for eco-smart tourism ventures.

**Table 6:** Findings of mediating effect.

Hypothesis	Indirect effect	Indirect Effect (β) (95% Confidence Interval)	Findings
*H10*: AR-A --> TE --> DT	*Path 1 (AR-A–> TE)*: β = 0.42, p-value = 0.000, z-value = 4.878	Β = 0.20 (CI = 0.14, 0.29)	Complementary mediation
	*Path 2 (TE–> DT)*: β = 0.47, p-value = 0.000, z-value = 4.942		

## Discussion

The findings of this study provide meaningful insights into how AR adoption contributes to the digital transformation of eco-smart tourism ventures, with Tourist Engagement playing a pivotal mediating role. Overall, the results underscore that AR is not only a technological tool but also a strategic enabler for sustainability, cultural enrichment, and innovation in the tourism sector. By integrating immersive and interactive digital elements into tourism experiences, small tourism ventures in Malaysia can enhance their competitiveness, strengthen their green brand image, and respond more effectively to the increasing demands for sustainable and digitally enabled travel experiences. This aligns with the growing body of research suggesting that digital technologies serve as catalysts for transformation in the tourism and hospitality industry^124^; Buhalis et al. 2024^125^).

From a theoretical perspective, this study advances the S-O-R framework by extending its application into the domain of AR driven eco-smart tourism transformation within emerging market SMEs. Specifically, the study enriches the organism component by conceptualizing it as a multi-dimensional construct that captures cognitive and affective tourist states, including immersive experience, environmental awareness support, cultural enrichment, trust, perceived sustainability value, and innovation orientation. This nuanced operationalization moves beyond traditional single dimensional interpretations of the organism stage and demonstrates how multiple experiential and perceptual responses can be simultaneously activated by a technological stimulus such as AR adoption. Furthermore, the study extends the response dimension by linking these organism level states to a higher order organizational outcome, namely digital transformation, thereby bridging individual level tourist experiences with firm level strategic transformation. In doing so, the study offers an integrated micro to macro perspective within the S-O-R logic, highlighting how technology induced tourist responses can translate into measurable organizational and sustainability outcomes. While the extension remains grounded within the original S-O-R structure, it provides empirical elaboration and contextual enrichment by demonstrating its applicability in technology enabled, sustainability-oriented tourism ecosystems.

The findings confirmed that AR adoption exerts a positive and significant influence on immersive experience (β = 0.29, *p* < .001, z = 3.791, f² = 0.132), thereby supporting Hypothesis 1. This result indicates that the implementation of AR technologies enhances the level of immersion experienced by tourists, particularly through increased presence, interactivity, and sensory engagement. In other words, rather than immersive experience driving adoption, the findings demonstrate that AR functions as a technological stimulus that actively generates richer and more engaging experiential outcomes. This is consistent with prior research showing that AR applications significantly enhance users’ sense of immersion and experiential value in tourism and hospitality contexts^48,68^. Furthermore, existing studies have emphasized that AR enabled immersion strengthens both cognitive and emotional engagement, making tourism experiences more memorable and impactful^126^. Therefore, this study highlights that eco smart tourism ventures can enhance tourist experiences by strategically adopting AR technologies that prioritize immersive features, ultimately contributing to greater engagement and value creation.

The results confirmed that AR adoption has a positive and significant influence on environmental awareness support (EAS) (β = 0.31, *p* < .001, z = 3.904, f² = 0.163), thus supporting Hypothesis 2. This finding suggests that the implementation of AR technologies enhances tourists’ environmental awareness by enabling interactive visualization of eco-friendly practices, sustainability impacts, and conservation knowledge. Rather than environmental awareness driving adoption, the results indicate that AR serves as a technological stimulus that actively facilitates environmental learning and promotes responsible tourist behaviour. This is consistent with prior studies which emphasize that AR applications can effectively communicate sustainability information and strengthen tourists’ cognitive engagement with environmental issues^21,127^. Furthermore, scholars have argued that integrating sustainability-oriented features within AR solutions not only enriches the tourism experience but also strengthens eco conscious branding and value creation for tourism operators^128^. Therefore, this study highlights the role of AR as a strategic enabler of environmental awareness, supporting eco smart tourism ventures in aligning digital innovation with sustainability objectives.

The findings revealed that AR adoption has a positive and significant relationship with cultural enrichment (CE) (β = 0.34, *p* < .001, z = 4.227, f² = 0.179), thereby supporting Hypothesis 3. This suggests that the implementation of AR technologies enhances tourists’ cultural experiences by enabling more interactive, engaging, and contextually meaningful encounters with heritage, traditions, and local culture. Rather than cultural enrichment driving adoption, the results indicate that AR functions as a technological stimulus that actively facilitates cultural learning and appreciation. Through features such as virtual reconstructions of historical sites, storytelling overlays, and gamified cultural exploration, AR provides an innovative avenue for preserving and communicating cultural identity. This finding is consistent with prior studies which reported that AR enhances cultural appreciation and learning by allowing tourists to experience heritage in more immersive and personalized ways^129,130^. Furthermore, researchers have highlighted that AR driven cultural experiences foster stronger emotional connections between tourists and destinations, thereby enhancing overall experiential value^131^. Thus, the current study reinforces the argument that AR adoption in eco smart tourism serves as a strategic mechanism for promoting cultural sustainability and heritage conservation through enriched tourist experiences.

The results indicate that AR adoption does not have a significant relationship with trust (TR) (β = 0.03, *p* = .182, z = 1.135, f² = 0.009), leading to the rejection of Hypothesis 4. This finding suggests that the implementation of AR technologies does not automatically enhance tourists’ trust within eco smart tourism contexts. While AR is effective in generating immersive and engaging experiences, it appears insufficient in building perceptions of reliability, credibility, or security. This implies that trust is not inherently derived from technological features alone but may depend on broader factors such as service quality, organizational reputation, and the specific trust target, whether trust in the technology itself or in the tourism provider. This finding aligns with prior research indicating that trust tends to be more critical in high risk or transaction-oriented contexts, such as e commerce or data sensitive environments, rather than in experiential and hedonic domains like tourism^132,133^. Furthermore, studies suggest that while AR enhances experiential value, trust formation requires consistent service delivery and transparency beyond digital augmentation^134^. Therefore, the current result highlights that although AR adoption contributes to experiential and sustainability related outcomes, its role in fostering trust remains limited, indicating that tourism ventures must complement AR implementation with broader trust building strategies.

The results confirmed that AR adoption has a strong and significant relationship with perceived sustainability value (PSV) (β = 0.38, *p* < .001, z = 4.536, f² = 0.186), thereby supporting Hypothesis 5. This finding underscores that the implementation of AR technologies enhances tourists’ perceptions of sustainability by enabling interactive and informative experiences that highlight environmental responsibility and eco-friendly practices. Rather than perceived sustainability value driving adoption, the results indicate that AR functions as a technological stimulus that actively shapes sustainability related perceptions. Through features such as virtual eco tours, digital storytelling, and visualization of environmental impacts, AR allows tourists to better understand and appreciate sustainable tourism practices. This is consistent with prior research emphasizing that digital technologies can strengthen sustainability perceptions by making environmental value more visible and experiential^135^. Moreover, AR’s ability to reduce reliance on physical resources and support cultural and environmental preservation reinforces its role as a green technology enabler^136^. The relatively strong effect size (f² = 0.186) further indicates that AR driven sustainability perceptions represent a meaningful outcome, highlighting that eco smart tourism ventures can leverage AR not only to enhance experiences but also to strengthen their sustainability positioning and value creation.

The findings revealed that AR adoption has a significant and positive relationship with innovation orientation (IO) (β = 0.25, *p* = .001, z = 3.468, f² = 0.117), thereby supporting Hypothesis 6. This suggests that the implementation of AR technologies enhances the innovation orientation of eco smart tourism ventures by encouraging experimentation, creativity, and openness to new ideas. Rather than innovation orientation driving adoption, the results indicate that AR acts as a technological stimulus that fosters a more proactive and innovation driven mindset within tourism ventures. By introducing immersive and interactive capabilities, AR enables businesses to explore novel ways of delivering value, redesign service offerings, and differentiate themselves in competitive markets. This finding is consistent with prior research highlighting that the adoption of advanced digital technologies can stimulate innovation by enabling new forms of value creation and service enhancement^137,138^. In the tourism context, AR provides opportunities to reimagine visitor experiences, integrate sustainability with digital solutions, and support eco smart practices, thereby reinforcing innovation driven transformation^139^. The moderate effect size (f² = 0.117) indicates that while AR driven innovation orientation is not the strongest outcome, it remains a meaningful pathway through which digital technologies contribute to the strategic development of eco smart tourism ventures.

The results confirmed that AR adoption has a significant and positive influence on the digital transformation of eco smart tourism ventures (β = 0.19, *p* = .003, z = 3.051, f² = 0.078), thereby supporting Hypothesis 7. Although the effect size is modest, the findings highlight AR as a meaningful enabler in advancing the digitalization agenda of small tourism ventures. By integrating AR technologies into service delivery and visitor experiences, tourism enterprises are able to modernize their operations, enhance customer engagement, and strengthen their competitive positioning in the digital economy. Importantly, the digital transformation examined in this study is primarily perceptual in nature, capturing how tourism ventures perceive improvements in innovation, branding, and customer experience, rather than directly measuring structural transformation outcomes such as process automation, operational efficiency, or revenue diversification. This distinction suggests that AR adoption may initially contribute more strongly to symbolic transformation, such as enhancing innovation image and digital branding, while its impact on deeper structural transformation may require longer term investment, organizational change, and integration with core business processes. This outcome is consistent with previous research indicating that AR adoption accelerates digital transformation by fostering innovative business models and enabling real time, technology driven customer interactions^140,141^. In the context of eco-smart tourism, AR adoption not only supports digital competitiveness but also aligns with sustainable practices by reducing reliance on physical resources and creating more engaging, low impact visitor experiences. Therefore, the findings highlight that AR driven digital transformation in eco-smart tourism ventures should be understood as a staged process, beginning with perceptual and experiential enhancements and progressively advancing toward more structural and operational changes. Hence, these results underscore the role of AR adoption as a catalyst for eco-smart tourism ventures seeking to transition toward a digitally enabled and sustainability driven future.

The findings revealed that augmented reality (AR) adoption has a strong and positive influence on tourist engagement (β = 0.42, *p* < .001, z = 4.878, f² = 0.189), thereby supporting Hypothesis 8. This result demonstrates that the integration of AR technologies significantly enhances how tourists interact with and experience eco-smart tourism ventures. Through interactive storytelling, real-time information overlays, and immersive content, AR creates memorable and personalized experiences that deepen visitor involvement and satisfaction. This aligns with earlier studies that emphasized AR’s capacity to foster higher levels of engagement by offering experiential value and co-creating meaningful encounters between tourists and destinations^38,130^. Furthermore, prior research highlights that AR-driven engagement extends beyond the visit itself, encouraging positive word-of-mouth, repeat visits, and long-term loyalty^45^. In the context of eco-smart tourism, the ability of AR to stimulate tourist engagement is particularly crucial, as it allows businesses to simultaneously promote sustainability awareness and enhance customer experience. Thus, these findings underscore AR adoption as a vital driver for cultivating engaged, informed, and environmentally conscious tourists.

The results confirmed that tourist engagement has a strong and positive influence on the digital transformation of eco-smart tourism ventures (β = 0.47, *p* < .001, z = 4.942, f² = 0.195), thereby supporting Hypothesis 9. This indicates that when tourists are actively engaged—through interactive, personalized, and immersive experiences facilitated by AR technologies—businesses are more likely to accelerate their digital transformation processes. Engaged tourists not only demand more sophisticated digital experiences but also provide valuable feedback, user-generated content, and behavioural insights that drive innovation in business operations. This finding is consistent with prior studies that emphasize the pivotal role of customer engagement in shaping digital transformation strategies within service-oriented industries^142,143^. Moreover, tourist engagement contributes to co-creation of value, enhancing both customer satisfaction and organizational learning, which are crucial enablers of sustainable digital transformation^144^. In the context of eco-smart tourism, engaged tourists can act as catalysts for adopting advanced technologies, ensuring that digital transformation aligns with both environmental objectives and market expectations. Thus, these findings highlight the central role of tourist engagement as a strategic bridge between AR adoption and the broader digital transformation of tourism ventures.

The findings provided strong evidence for Hypothesis 10, which posited that tourist engagement mediates the relationship between AR adoption and the digital transformation of eco-smart tourism ventures. The results revealed that the direct path from AR adoption to tourist engagement was significant (β = 0.44, *p* < .001, z = 4.894), as was the path from tourist engagement to digital transformation (β = 0.48, *p* < .001, z = 4.961). This establishes a complementary mediation effect, confirming that while AR adoption directly contributes to digital transformation, its influence is substantially amplified when mediated through tourist engagement. In other words, AR technologies alone can stimulate digital transformation, but their full transformative potential is realized only when tourists are actively engaged with the immersive and interactive experiences they create. These findings align with prior studies that highlight the mediating role of user or customer engagement in digital innovation and transformation processes^145,146^. In the tourism sector, engagement fosters co-creation of value, enhances trust in digital solutions, and encourages tourists to participate in shaping digitally enhanced services. Moreover, the mediation effect emphasizes that eco-smart tourism ventures cannot rely solely on technology adoption; rather, they must design AR applications that meaningfully engage tourists to drive sustainable digital transformation. This result underscores the importance of positioning tourist engagement as a strategic enabler that connects technology adoption with broader organizational change, ensuring that AR adoption translates into tangible business and sustainability outcomes.

## Implications

### Theoretical implications

This study contributes significantly to the theoretical understanding of technology adoption and digital transformation in eco-smart tourism ventures by extending the Stimulus–Organism–Response (S-O-R) framework. By conceptualizing augmented reality (AR) adoption as the stimulus, tourist engagement as the organism, and digital transformation as the response, the research provides an empirically validated model that explains how technology-driven stimuli influence both behavioural and organizational outcomes. This reinforces the suitability of S-O-R in explaining technology-mediated value creation in tourism.

First, the study demonstrates that AR adoption enhances immersive experiences, cultural enrichment, environmental awareness, sustainability perceptions, and innovation orientation, thereby expanding the theoretical boundaries of AR’s role in tourism beyond entertainment to encompass sustainability and transformation-oriented outcomes. Second, the research establishes tourist engagement as a mediating mechanism, which has been relatively underexplored in the tourism innovation literature. This finding highlights that digital transformation is not solely technology-driven but also socially and psychologically mediated through engagement, thereby adding a critical human dimension to digital transformation theory.

Third, the study enriches AR adoption research by showing its differential effects—while constructs like perceived sustainability value and cultural enrichment strongly link to AR adoption, trust does not, signalling boundary conditions in the AR–adoption relationship. This nuance contributes to the refinement of technology acceptance models in tourism contexts. Finally, by situating the research in eco-smart tourism ventures in Malaysia, the study expands the contextual understanding of digital transformation in small-scale tourism enterprises, a sector often overlooked in mainstream digital transformation theory.

### Practical implications

The findings provide several important insights for practitioners, policymakers, and tourism stakeholders aiming to foster eco smart tourism transformation through AR technologies. Given that the data were collected from tourism venture representatives, the implications are framed at the managerial and operational level, focusing on strategic decision making, resource allocation, and measurable organizational outcomes. Moreover, the direction of relationships validated in this study positions AR adoption as a primary driver of transformation, which should guide implementation priorities in practice.

First, the results show that AR adoption directly accelerates digital transformation while also indirectly enhancing it through tourist engagement. For practitioners, this underscores the importance of not only investing in AR technologies but also designing interactive, immersive, and emotionally engaging AR applications that connect tourists with cultural, environmental, and sustainability narratives. Given the relatively strong standardized effect of AR adoption on tourist engagement (β = 0.42), managers should prioritize engagement centric AR features such as interactive storytelling, gamified exploration, and real time personalization, as these are likely to yield the highest returns in terms of digital transformation outcomes. Tourism ventures should therefore move beyond simple AR trials and focus on integrated AR experiences that foster long term visitor engagement and measurable outcomes such as increased dwell time, repeat visitation, and positive digital reviews.

Second, the significant role of perceived sustainability value indicates that tourists are more likely to embrace AR enabled services when they perceive clear environmental and social benefits. Given its relatively strong effect size (f² = 0.186), managers should strategically position AR applications as sustainability communication tools that visibly demonstrate eco-friendly practices and conservation outcomes. Practically, eco smart tourism ventures should highlight how AR solutions reduce environmental impact, such as replacing printed guides with digital storytelling, and incorporate features that educate tourists on responsible behaviour, thereby strengthening both brand positioning and customer loyalty.

Third, the findings suggest the need for tiered implementation strategies based on the technological readiness and resource capacity of SMEs. For low capacity SMEs with limited financial and technical resources, AR adoption can begin with low cost and scalable solutions such as mobile based AR applications, QR enabled storytelling, or partnerships with third party AR developers. These entry level strategies minimize infrastructure investment while still enabling experiential enhancement. In contrast, high capacity SMEs with greater digital maturity can invest in advanced AR ecosystems, including immersive applications, integrated booking systems, and data analytics capabilities that track user engagement and conversion metrics. Such differentiation ensures that AR adoption remains feasible and strategically aligned with firm capabilities rather than imposing uniform expectations across heterogeneous SMEs.

Fourth, the findings reveal that innovation orientation is strengthened by AR adoption, which implies that small tourism businesses should treat AR not merely as a digital tool but as a strategic enabler of innovation capability. Training programs, digital literacy initiatives, and collaborative ecosystems between AR developers, SMEs, and tourism boards could accelerate the diffusion of AR technologies across the sector. Managers should view AR implementation as part of a broader organizational transformation process that fosters continuous innovation, rather than as a one-time technological upgrade.

Finally, the results offer policy level implications. Policymakers and tourism authorities in Malaysia and similar emerging economies should develop supportive digital ecosystems, including funding schemes, capacity building workshops, and public private partnerships, to encourage AR adoption among SMEs. Policy interventions should also be tiered, offering basic digital support for early stage SMEs while facilitating advanced innovation grants and infrastructure development for more digitally mature firms. By embedding AR into eco smart tourism, destinations can differentiate themselves globally, strengthen sustainability branding, and achieve long term competitiveness in the digital tourism economy.

## Limitations and recommendations

Although this study provides valuable contributions to understanding the adoption of AR and its role in the digital transformation of eco-smart tourism ventures, certain limitations should be acknowledged to guide interpretation and future research directions. First, the research applied a cross sectional design, which restricts the ability to establish causal relationships over time. While the findings confirm significant associations between AR adoption, tourist engagement, and digital transformation, the dynamic nature of technology adoption suggests that longitudinal studies would be better suited to capture how these relationships evolve as businesses and tourists gain more experience with AR tools.

Second, the reliance on self-reported survey data may introduce social desirability bias or common method variance, despite statistical checks indicating acceptable levels. Although procedural remedies were applied, the potential for common method bias cannot be entirely ruled out in cross sectional designs where data are collected from a single source. Future research is encouraged to adopt multi source and multi respondent approaches, for instance by collecting data from both tourists and tourism managers, to enhance data robustness and reduce single source bias.

Third, the study may be subject to potential endogeneity concerns, particularly regarding the relationship between innovation orientation and AR adoption. While AR adoption is conceptualized as an exogenous construct in this study, it is plausible that innovation-oriented ventures are more inclined to adopt AR, indicating possible reciprocal or bidirectional relationships. Future research should address this limitation by employing longitudinal designs, instrumental variable approaches, or experimental methods to better establish causal directionality and mitigate endogeneity bias.

Third, the study focused on small tourism ventures across seven states in Malaysia, which, while providing a valuable localized perspective, may limit generalizability to other national or regional contexts. In particular, the cultural, institutional, and economic specificities of Malaysian SMEs, such as varying levels of digital readiness, government support, and societal attitudes toward technology, may influence the observed relationships. Future studies should incorporate cross cultural comparisons to examine whether the proposed relationships hold across different institutional and cultural environments.

Lastly, the model primarily examined internal factors such as immersive experience, environmental awareness, and innovation orientation. However, external enablers and constraints, including policy incentives, digital ecosystem readiness, and tourists’ prior technological familiarity, were not included. Integrating these contextual elements in future studies could provide a more holistic understanding of AR driven transformation.

Building on these limitations, future research should adopt more advanced and integrative methodological approaches. Specifically, longitudinal designs are recommended to capture the dynamic and evolving nature of digital transformation over time. In addition, future studies should incorporate behavioural and objective metrics, such as AR application usage data, booking conversions, and online review analytics, to triangulate perceptual findings with actual user behaviour. Such triangulation would significantly enhance the validity and practical relevance of the findings, particularly in understanding how AR driven engagement translates into measurable business outcomes.

Despite these limitations, the study offers several practical and theoretical recommendations. For researchers, there is a strong need to extend the current framework by incorporating macro level factors such as government regulations, funding mechanisms, and industry collaborations, alongside micro level variables like organizational readiness and user trust. Additionally, testing the model in diverse geographical and cultural contexts would enhance external validity and identify universal versus context specific drivers of AR adoption.

For practitioners, the findings highlight the importance of designing AR applications that not only entertain but also enrich cultural experiences, promote environmental awareness, and demonstrate sustainability value. Eco smart tourism ventures are encouraged to strategically view AR adoption as a tool for innovation orientation and digital competitiveness, rather than as an optional enhancement. To maximize benefits, businesses should invest in staff training and digital capacity building, ensuring seamless integration of AR into their services.

## Data Availability

Data are available from the corresponding author upon reasonable request due to privacy restrictions.
